# Impact of ventilatory and laboratory parameter trajectories on short-term survival in acute respiratory distress syndrome patients: a retrospective study using joint models

**DOI:** 10.1186/s40001-025-02650-z

**Published:** 2025-05-20

**Authors:** Lars Heubner, Paul Leon Petrick, Evelyn Trips, Andreas Güldner, Maximillian Ragaller, Martin Mirus, Martin Scharffenberg, Axel Rand, Oliver Tiebel, Thea Koch, Peter Markus Spieth

**Affiliations:** 1 Department of Anesthesiology and Intensive Care Medicine, Faculty of Medicine and University Hospital Carl Gustav Carus, TUD Dresden University of Technology, Dresden, Germany; 2 Coordination Centre for Clinical Trials, Faculty of Medicine Carl Gustav Carus, TUD Dresden University of Technology, Dresden, Germany; 3 Institute of Clinical Chemistry, Faculty of Medicine and University Hospital Carl Gustav Carus, TUD Dresden University of Technology, Dresden, Germany

**Keywords:** Respiratory distress syndrome, Pneumonia, Disease progression, Respiration, Artificial, Survival analysis

## Abstract

**Background:**

Clinical research is based on the parameters at defined time points, such as admission, diagnosis or discharge, for the purpose of risk factor analysis in relation to outcome. However, these parameters are collected with greater frequency in clinical practice. The objective of this study was to demonstrate a correlation between the time course of closely monitored parameters, such as blood gases, ventilatory parameters or routine laboratory values, and the survival of patients with acute respiratory distress syndrome (ARDS) caused by pneumonia.

**Methods:**

This single-center, retrospective study included 274 ARDS patients with primary pneumonia requiring invasive mechanical ventilation. Patients were treated at a German university hospital between January 2014 and April 2021. Ethical approval was obtained from the local ethics committee (BO-EK-374072021). Longitudinal data on ventilatory and inflammatory parameters were collected during ICU stays. The analysis was conducted using descriptive statistics, cox regression and joint models. Joint modelling was used to integrate the progression of these parameters with survival outcomes, with the modelling of longitudinal data performed using quadratic B-splines.

**Results:**

The cohort included 274 patients, with an ICU mortality rate of 49.6%. Non-survivors were older (67 vs. 62 years, *p* < 0.001) and had higher SOFA scores at admission (10 vs. 8, *p* < 0.001). Differences in ventilatory parameters, including driving pressure and the PaO₂/FIO₂ ratio, as well as inflammatory markers such as procalcitonin, were observed between survivors and non-survivors during the ICU stay. The joint model analysis revealed a significant effect of the time course of parameters, such as positive end-expiratory pressure (PEEP), peak airway pressure (Ppeak), driving pressure, minute ventilation, tidal volume, C-reactive protein (CRP) and procalcitonin on mortality. The increase over time (slope-dependent association) for these parameters was strongly associated with mortality. For example, driving pressure was associated with mortality both by its current value (HR 1.16) and by its increase over time (HR 7.10). Similarly, tidal volume (HR 0.72 and 0.07), minute ventilation (HR 0.91 and 0.36), PEEP (HR 1.32 and 13.52), Ppeak (HR 1.20 and 3.28) and CRP (HR 1.14 and 4.25) showed a current value association and a strong slope-dependent association with mortality.

**Conclusion:**

This study underscores the importance of analyzing the dynamics of clinical parameters rather than static values for ARDS management. The findings suggest that changes in routine clinical parameters over time provide valuable prognostic information and should be prioritized in risk assessment and therapeutic decision making.

**Supplementary Information:**

The online version contains supplementary material available at 10.1186/s40001-025-02650-z.

## Background

Acute respiratory distress syndrome (ARDS) is common in intensive care units (ICU) [1]. The pathophysiology of ARDS is characterised by complex interactions between inflammation, lung parenchymal injury and gas exchange, which collectively contribute to a high morbidity and mortality [2].

Pneumonia represents one of the most common causes of ARDS [3, 4]. Particularly, during the course of the coronavirus pandemic, the number of pneumonia-related ARDS has increased even more. The objective of the majority of retrospective ARDS studies was to identify specific risk or prognostic factors associated with mortality. These risk or prognostic factors were typically selected at a specific time point. For example, Azoulay et al. [5] demonstrated the correlation between pre-existing conditions, including chronic respiratory disease and chronic heart failure, and an increased risk of mortality within the first 28 days. Furthermore, the relationship between the invasiveness of mechanical ventilation as indicated by parameters such as driving pressure or mechanical power, and the outcome of ARDS patients has been already described [6–8].

However, clinical experience suggests, that it is not the values at a specific time point that are most relevant for the outcome, but rather the dynamics of the values. The analysis of clinical parameters at different times of the ICU stay was only conducted in a limited number of studies [9–11].

There are different ways of assessing the course of a clinical parameter and its effect on outcome. Joint modelling has already been used in various studies and medical questions [12–14]. Joint models comprise two submodels. Firstly, it is necessary to model the longitudinal data collected and its development. Secondly, it is necessary to model the time until the occurrence of a certain event [15], which is often the death or discharge of a patient.

Given the potential for close-meshed, continuous data collection in invasively ventilated patients in intensive care units, the use of joint models is a reasonable approach. The objective of this study is to show the influence of dynamic changes in parameters on mortality and to discuss possible advantages of this approach.

## Methods

### Study design

We performed a single-center, retrospective study at a German university hospital (University Hospital “Carl Gustav Carus” at Dresden University of Technology). All mechanically ventilated patients with ARDS caused by pneumonia fulfilling the Berlin criteria [2] between January 2014 and April 2021 were included in this study. Patients with aspiration pneumonia or other causes of ARDS were excluded. Preselection was carried out with automatic selection according to International Statistical Classification of Diseases and Related Health Problems (ICD) coding.

### Data collection and outcome definitions

The primary outcome was defined as the time from ICU admission to death from any cause in the ICU. Patients who were still alive at ICU discharge were censored.

In order to gain insight into the clinical time course, parameters of ventilation and inflammation have been selected based on their clinical relevance. Respiratory rate, minute ventilation, tidal volume per kilogram of ideal body weight, driving pressure, ratio of partial pressure of oxygen and inspiratory oxygen fraction (PaO₂/F_I_O₂ ratio), positive end-expiraory pressure (PEEP), peak airway pressure (Ppeak), C-reactive protein (CRP), leucocytes and procalcitonin were subjected to analysis. The ventilatory parameters (respiratory rate, minute ventilation, tidal volume per kilogram of ideal body weight, driving pressure, PEEP, Ppeak) were collected on an hourly basis by the medical staff within the patient data management system from the time of admission to the time of discharge from the ICU. The PaO₂/FIO₂ ratio was measured every 4 h by means of blood gas analysis. Laboratory parameters, namely CRP, procalcitonin and leukocytes, were assessed at least once per day. In instances where multiple measurements were recorded on a single day, the mean was calculated for each parameter. Specific indices, such as the Charlson Comorbidity Index (CCI) or the Sequential Organ Failure Assessment (SOFA) score, were recorded on the day of ICU admission. The treatment protocols are in strict adherence to the institutional standard operating procedures (SOPs) based on the actual guidelines, particularly those pertaining to ventilation, anticoagulation, and extracorporeal membrane oxygenation (ECMO) therapy.

### Statistical analysis

Statistical analyses were performed using SPSS Statistics 27 software (IBM, Inc., Armonk, NY, USA) and SAS software, version 9.4, of the SAS System for Windows (SAS Institute, Inc., 2023). All categorical variables are described as absolute and relative frequencies; comparisons between groups were performed via Fisher's exact test. Continuous variables are presented as medians along with lower and upper quartiles and ranges; group comparisons were based on the Mann‒Whitney *U* test. Skewed data were logarithmically transformed to base 2 (Log2) (PaO_2_/FIO_2_ ratio, procalcitonin) or square root (CRP, leukocytes) values. The significance level was set at *α* = 0.05. As this is an explorative analysis, no adjustment for multiple testing was performed.

Univariate unadjusted Cox regression analyses were employed to examine the association between an independent variable and ICU mortality.

We utilized the previously published SAS macro %JM to estimate a joint model for each parameter separately [16]. The modelling of the longitudinal data for the joint models was performed via quadratic B-splines. Each model was adjusted for SARS-CoV-2 infection status, sex, age, body mass index (BMI) and SOFA score at admission without the Glasgow Coma Scale (GCS).

### Ethics

The study was designed and performed in accordance with the Declaration of Helsinki. The institutional Ethics Committee approved the study protocol (BO-EK-374072021). The Strengthening the Reporting of Observational Studies in Epidemiology (STROBE) Statement: Guidelines for reporting observational studies were followed [17].

## Results

### Characteristics of the cohort

**Fig. 1:**
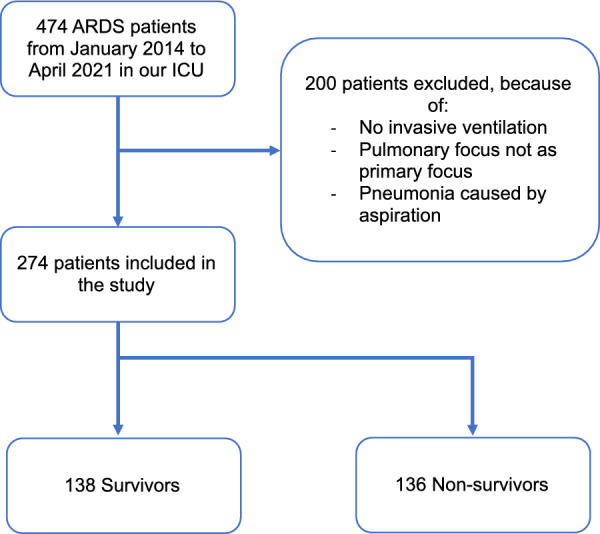
Flowchart. ARDS: acute respiratory distress syndrome; ICU: intensive care unit

A total of 274 patients were included in this study. The Consort diagram is shown in Fig. 1. The ICU mortality was 49.6% (136/274).

Table 1 presents an overview of the demographic data and baseline characteristics of the patients, with supplementary information provided in Table e1 (Additional file 1). There was a greater percentage of male patients among non-survivors (78.7%) than among survivors (67.4%; *p* = 0.041). Non-survivors were older than survivors were (67 vs. 62 y; *p* < 0.001). There was no significant difference in the median BMI between non-survivors and survivors (27.7 kg/m^2^ vs. 29.4 kg/m^2^).

**Table 1:** Demographic and baseline characteristics

	Non-survivor	Range	Survivor	Range	*p*
N	136		138		
Male	107 (78.7%)		93 (67.4%)		*0.041*
Age [years]	67 (59; 73)	33–92	62 (55; 69)	20–83	< *0.001*
Body mass index [kg/m^2^]	27.7 (25.0; 31.0)	17.3–70.3	29.4 (25.4; 33.4)	18.9–66.8	*0.085*
COVID-19	93 (68.4%)		90 (65.2%)		*0.609*
Direct transfer to our ICU from other hospital	97 (71.3%)		98 (71.0%)		*1.000*
Charlson comorbidity index	4 (2; 6)	0–11	3 (2; 5)	0–11	*0.008*
Arterial Hypertension	93 (68.4%)		97 (70.3%)		*0.794*
Cardiovascular disease	37 (27.2%)		31 (22.5%)		*0.403*
Neurovascular symptoms	15 (11.0%)		12 (8.7%)		*0.549*
Thrombembolic events in medical history	11 (8.1%)		5 (3.6%)		*0.130*
Chronic arrhythmias	35 (25.7%)		25 (18.1%)		*0.145*
COPD	10 (7.4%)		11 (8.0%)		*1.000*
Other pulmonary disease	11 (8.1%)		8 (5.8%)		*0.485*
Nicotine abuse	16 (11.8%)		27 (19.6%)		*0.096*
Diabetes mellitus	51 (37.5%)		57 (41.3%)		*0.539*
Previous organ or bone marrow transplantation	4 (2.9%)		6 (4.3%)		*0.749*
Chronic renal failure	18 (13.2%)		12 (8.7%)		*0.251*
Chronic need of renal replacement therapy	7 (5.1%)		1 (0.7%)		*0.035*
ACE inhibitors	25 (19.5%)		39 (31.2%)		*0.043*
AT2 receptor blocker	34 (26.6%)		28 (22.4%)		*0.468*
Beta blocker	60 (46.2%)		56 (44.8%)		*0.900*
Antithrombotic drug	39 (30.5%)		28 (22.2%)		*0.155*
DOAC	24 (18.8%)		19 (15.1%)		*0.504*
Corticosteroids	16 (12.5%)		14 (11.1%)		*0.846*
Immunosuppressive Drugs	13 (10.1%)		6 (4.8%)		*0.152*
Nosocomial infection	26 (19.1%)		16 (11.6%)		*0.095*

Data are median (Interquartile range) or n (%). *ACE* Angiotensin-converting enzyme; *AT2* Angiotensin II; *COPD* Chronic obstructive pulmonary disease; *DOAC* Direct oral anticoagulants; *ECMO* extracorporeal membrane oxygenation; *ICU* Intensive care unit

No significant differences were observed between survivors and non-survivors regarding their pre-existing conditions. Only the need for chronic renal replacement therapy was significantly higher in non-survivors (5.1% vs. 0.7%; *p* = 0.035).

With regard to medication, angiotensin-converting enzyme (ACE) inhibitors were used less frequently among non-survivors than among survivors (*p* = 0.043). No significant differences were observed in the use of angiotensin II (AT2) receptor blockers, beta blockers, antithrombotic drugs, direct oral anticoagulants (DOACs), corticosteroids, or immunosuppressive drugs between groups.

The most prevalent pneumonia pathogen was identified as SARS-CoV-2 (183/274, 66.8%). According ICD-10 coding, the subsequent diagnoses were influenza virus (9/274, 3.3%), Legionella (7/274, 2.6%) and Streptococcus pneumoniae (6/274, 2.2%).The incidence of nosocomial infections was not significantly different between non-survivors and survivors (19.1% vs. 11.6%, *p* = 0.095).

Some of ICU characteristics are shown in Table 2. When compared with survivors, non-survivors had shorter ICU stays (median stay 13 days vs. 16 days; *p* = 0.015). There was a significantly increased prevalence of septic shock in non-surviving patients than in survivors (non-survivors: 29.4%; survivors: 15.4%; *p* = 0.009). Compared with survivors, non-surviving patients presented a lower pH at admission (7.36 vs. 7.39; *p* = 0.018). The SOFA score at ICU admission, excluding the GCS score, was also significantly higher in non-survivors (10 points) than in survivors (8 points) (*p* < 0.001). The maximum values of blood lactate and procalcitonin were significantly elevated in non-survivors when compared with survivors (*p* = 0.001 and *p* < 0.001).

**Table 2:** ICU characteristics

	Nonsurvivor	Range	Survivor	Range	*p*
N	136		138		
ARDS mild at ICU admission	7 (5.1%)		7 (5.1%)		*1.000*
ARDS moderate at ICU admission	60 (44.1%)		51 (37.0%)		*0.268*
ARDS severe at ICU admission	69 (50.7%)		77 (55.8%)		*0.468*
Septic shock at ICU admission	40 (29.4%)		21 (15.4%)		*0.009*
Lowest Horovitz index at ICU	52.5 (45; 67.5)	22.5–135	75 (60; 90)	22.5–225	< *0.001*
P_mean_ at admission [mbar]	20 (18; 22)	8–30	19 (16; 22)	7–29	*0.180*
PEEP at admission [mbar]	14 (10; 15)	3–20	13 (10; 15)	5–20	*0.597*
pH at admission	7.36 (7.30; 7.42)	6.81–7.59	7.39 (7.32; 7.44)	7.01–7.62	*0.018*
SOFA score at ICU admission without GCS	10 (8; 12)	5–18	8 (7; 10)	3–16	< *0.001*
Lactate at ICU admission [mmol/L]	1.5 (1.0; 2.4)	0.5–26.0	1.2 (0.9; 1.6)	0.4–12.3	*0.001*
Duration of mechanical ventilation in ICU [days]	13 (8; 18.5)	1–61	12 (7; 19)	2–89	*0.893*
Prone position	107 (78.7%)		109 (79.0%)		*1.000*
CRRT	79 (58.1%)		34 (24.6%)		< *0.001*
Duration of CRRT [hours]	146.4 (61.3; 281.5)	1.4–906.5	311.3 (170.2; 490.3)	0.7–1346.21	*0.001*
ECMO	59 (43.4%)		37 (26.8%)		*0.005*
Duration of ECMO [hours]	278.2 (163.3; 375.2)	16.8–858.6	256.9 (190.1; 343.5)	9.2–1068.3	*0.738*
NO inhalation	69 (50.7%)		22 (15.9%)		< *0.001*
Argatroban at any time on ICU	8 (5.9%)		12 (8.7%)		*0.487*
UFH at any time on ICU	130 (95.6%)		92 (66.7%)		< *0.001*
LMWH at any time on ICU	52 (38.2%)		113 (81.9%)		< *0.001*
Bacteremia	68 (50.0%)		46 (33.3%)		*0.007*
CRP maximum value [mg/l]	308.1 (222.1; 371.5)	58.5–618.0	238.9 (163.0; 333.3)	31.4–644.3	*0.001*
Leucocytes maximum value [GPt/L]	22.1 (17.0; 28.9)	1.1–73.2	18.7 (13.2–28.0)	3.7–94.7	< *0.001*
Leucocytes minimum value [GPt/L]	7.3 (4.9; 10.1)	0.0–22.8	6.9 (5.2; 9.3)	0.5–19.0	*0.618*
Procalcitonin maximum value [ng/ml]	7.4 (2.2; 16.5)	0.2–185.8	2.1 (0.6; 10.1)	0.1–397.1	< *0.001*
Duration of ANE-ICU stay [days]	13 (8; 19)	1–60	16 (9; 25)	1–89	*0.015*
Duration of stay at UKD [days]	14 (9; 20.5)	1–89	24 (15; 33)	3–93	< *0.001*

Data are median (Interquartile range) or n (%)

*ANE-ICU* Intensive care unit of the Department of Anesthesiology and Critical Care Medicine; *ARDS* Acute respiratory distress syndrome; *CRP* C-reactive protein; *CRRT* Continuous renal replacement therapy; *ECMO* extracorporeal membrane oxygenation; *ICU* Intensive care unit; *LMWH* Low-molecular-weight heparin; *NO* Nitric oxide; *PEEP* Positive end-expiratory pressure; *P*_*mean*_ Mean airway pressure; *SOFA* Sequential organ failure assessment; *SpO*_*2*_ Oxygen saturation; *UFH* Unfractionated heparin; *UKD* University hospital Dresden

When compared with survivors, non-surviving patients received continuous renal replacement (CRRT) (58.1% vs 24.6%; *p* < 0.001) and ECMO therapy (43.4% vs 26.8%, *p* = 0.005) more frequently.

Our data also revealed differences in the use of anticoagulants, with non-survivors more commonly receiving unfractionated heparin (UFH) (95.6% vs 66.7%; *p* < 0.001) and less commonly receiving low-molecular-weight heparin (LMWH) (38.2% vs. 81.9%; *p* < 0.001) compared to survivors.

Non-surviving patients had a significantly higher proportion of positive blood cultures indicating a secondary bacterial superinfection, with 50.0% (68/136) compared to survivors (46/138, 33.3%). The respective pathogens identified are listed in Table e1. Significant differences were found only for Staphylococcus aureus infection between survivors (2/138, 1.4%) and non-survivors (9/136, 6.6%; p = 0.034).

### Cox regression analysis of ICU mortality

The results of the univariate unadjusted Cox regression models are shown in Table 3. SARS-CoV-2 infection had a hazard ratio (HR) of 1.617 (confidence interval (CI) 1.117–2.340; *p* = 0.011). Older age was significantly associated with increased mortality (HR 1.039; CI 1.022–1.056; *p* < 0.001).

**Table 3:** Univariate unadjusted Cox regression

Variable	Unadjusted HR	CI	*p*
SARS-CoV 2 infection	1.617	1.117–2.340	*0.011*
Age	1.039	1.022–1.056	< *0.001*
Body mass index	1.002	0.980–1.025	*0.865*
Male	0.728	0.483–1.099	*0.131*
Arterial hypertension	1.009	0.703–1.449	*0.961*
Cardiovascular disease	1.120	0.767–1.635	*0.557*
Neurovascular symptoms	0.847	0.492–1.456	*0.547*
Coronary artery disease	0.814	0.494–1.341	*0.419*
Thrombembolic events in medical history	1.162	0.624–2.162	*0.636*
Chronic arrhythmias	1.161	0.787–1.710	*0.452*
COPD	0.901	0.473–1.718	*0.901*
Other pulmonary disease	1.246	0.671–2.313	*0.486*
Nicotine abuse	0.578	0.342–0.975	*0.040*
Diabetes mellitus	0.923	0.651–1.309	*0.653*
Chronic renal failure	1.222	0.742–2.011	*0.431*
Charlson comorbidity index	1.074	1.007–1.146	*0.030*
SOFA score at ICU admission without GCS	1.055	0.996–1.118	*0.070*
P_mean_ at admission	1.021	0.979–1.065	*0.338*
PEEP at admission	1.035	0.987–1.086	*0.157*
pH at admission	0.494	0.093–2.617	*0.407*
PaCO_2_ at admission	1.023	0.945–1.109	*0.569*
SpO_2_ at admission	0.979	0.954–1.005	*0.107*
Septic shock at ICU admission	1.294	0.894–1.874	*0.172*

Data are hazard ratio (HR) and 95% confidence interval (CI).: Chronic obstructive pulmonary disease; *CRRT* Continuous renal replacement therapy; *ECMO* extracorporeal membrane oxygenation; *GCS* Glasgow Coma Scale; *PaCO*_*2*_ partial pressure of carbon dioxide; *PEEP* Positive end-expiratory pressure; *P*_*mean*_ mean airway pressure; *SOFA* Sequential organ failure assessment; *SpO*_*2*_ Oxygen saturation

Nicotine abuse had a positive effect, with an HR of 0.578 (CI 0.342–0.975; *p* = 0.040). The Charlson comorbidity index had an HR of 1.074 (CI 1.007–1.146; *p* = 0.030).

Other factors, such as arterial hypertension, cardiovascular disease, and chronic renal failure, were not associated with the outcome studied.

### Clinical course parameters and joint models

**Fig. 2:**
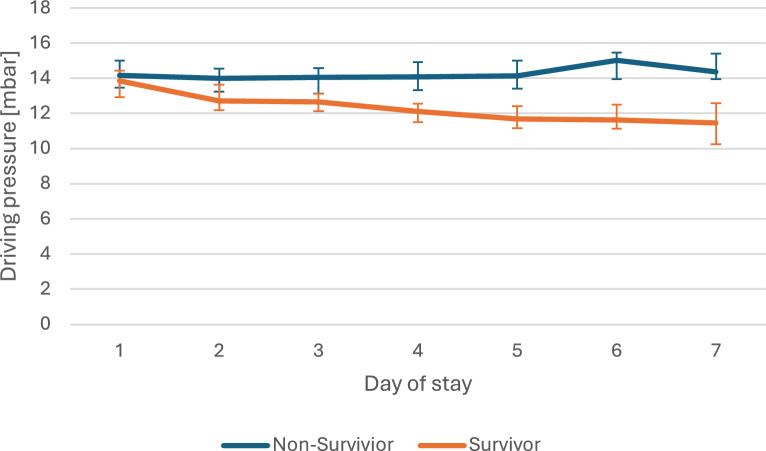
Clinical course of driving pressure. The data are presented as the median and its 95% CI of the daily values of nonsurvivors and survivors for the first seven days at intensive care unit

**Fig. 3:**
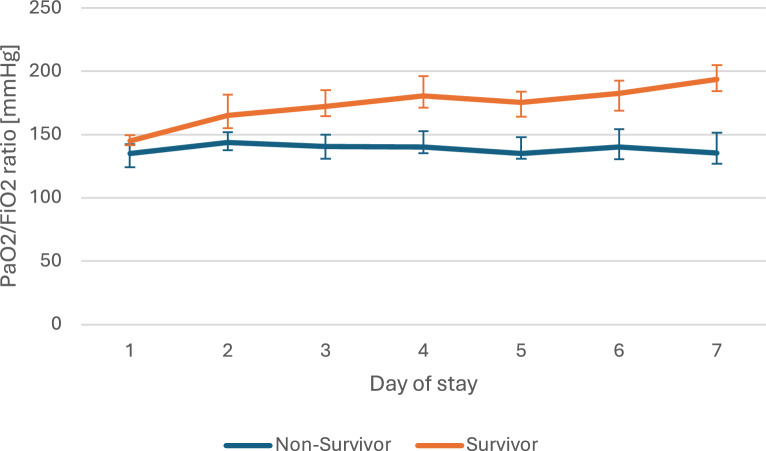
Clinical course of PaO_2_/FIO_2_ ratio. The data are presented as the median and its 95% CI of the daily values of non-survivors and survivors for the first seven days at intensive care unit

**Fig. 4:**
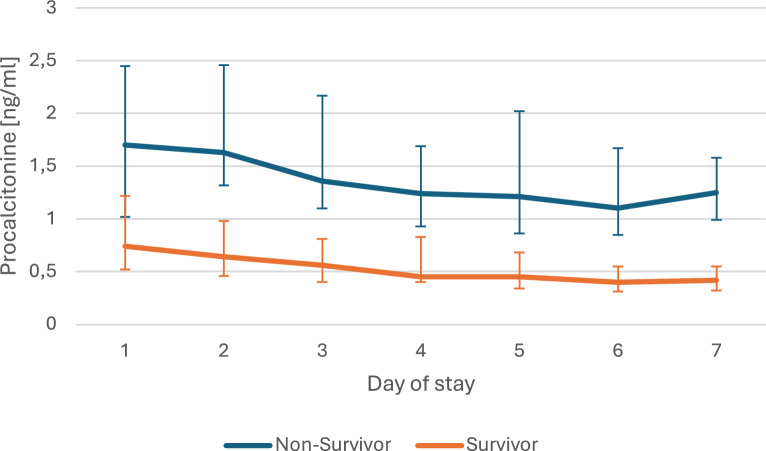
Clinical course of procalcitonin. The data are presented as the median and its 95% CI of the daily values of non-survivors and survivors for the first seven days at intensive care unit

The joint models used in this analysis integrate the trajectory of longitudinal data with the time to occurrence of an event. To illustrate the clinical course data, we have presented the median values for driving pressure, PaO_2_/F_I_O_2_ ratio and procalcitonin for every day of the stay, with a comparison between survivors and non-survivors in Figs. 2–4. As this is intended only for illustrative purposes, the diagrams were limited to 7 days of stay. The joint models included the complete course of all patients, which can be found in Tables e2–e11 (Additional file 1).

Figure 2 shows the course of the driving pressure in the first 7 days. It can be seen that the non-survivors had higher pressure values over the entire period. This difference is significant from day 4 onwards. In contrast to the non-survivors, a decrease in driving pressure can be seen in the survivors as the length of stay progresses. Figure 3 shows the course of the median PaO_2_/F_I_O_2_ ratio on the respective day of stay. While there is hardly any increase in the curve for non-survivors, the curve for survivors rises much more sharply in the period shown. The difference between the two curves is significant from day 2 of the stay. In Fig. 4, which shows the procalcitonin on the respective day of stay, the difference between survivors and non-survivors is also significant in the period shown from the second day onwards. However, both curves show a downward trend.

**Fig. 5:**
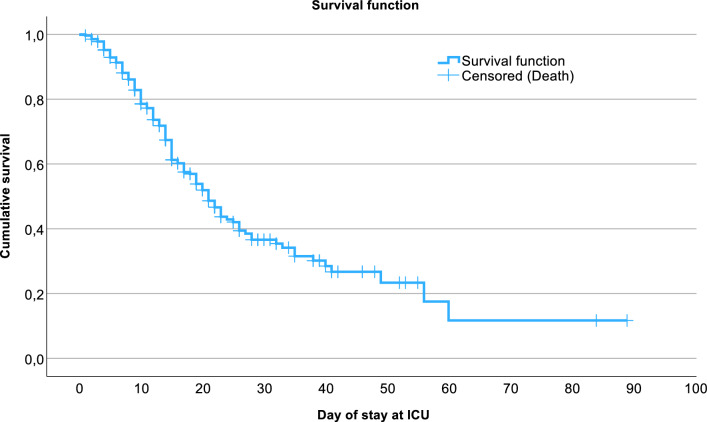
Kaplan–Meier curve of time to ICU mortality for the entire cohort. ICU: intensive care unit

To gain insight into the time to ICU mortality or discharge, a Kaplan–Meier curve of the cohort is presented in Fig. 5.

The results of the joint models are presented in Table 4. The joint model estimates two values. Firstly, a “current value association” is indicated, which signifies the risk associated with an increase of one unit in the value of the analysed parameter at a particular time point. Secondly, the model estimates a “slope-dependent association”, which indicates the risk of increasing the rate of change (slope) of the parameter's trajectory by one unit. The estimated regression parameters (current value association and slope-dependent association) were converted into a hazard ratio. A significant correlation between the outcome and the current value association was demonstrated in the joint model for all analysed parameters, with the exception of leukocytes. No significant correlation was demonstrated for the slope-dependent association of respiratory rate and leukocytes.

**Table 4:** Joint models

Parameter	Association (current-value)	Lower 95 CI	Upper 95 CI	HR	*p*
Respiratory rate [/min]	0.06866	0.02639	0.1109	1.07	0.0015
Minute ventilation [L]	− 0.09818	− 0.1520	− 0.04436	0.91	0.0004
Tidal volume [ml/kg of ideal bodyweight]	− 0.3264	− 0.4417	− 0.2111	0.72	< 0.0001
Driving pressure [mbar]	0.1513	0.09472	0.2078	1.16	< 0.0001
Log_2_ of Horovitz index [mmHg]	− 2.1077	− 2.6162	− 1.5992	0.12	< 0.0001
Log_2_ of Procalcitonin [ng/ml]	0.3325	0.2293	0.4358	1.39	< 0.0001
PEEP [mbar]	0.2811	0.1913	0.3709	1.32	< 0.0001
P_peak_ [mbar]	0.1837	0.1294	0.2380	1.20	< 0.0001
CRP (sqrt) [mg/l]	0.1312	0.07443	0.1880	1.14	< 0.0001
Leucocytes (sqrt) [GPt/L]	− 0.02325	− 0.1928	0.1462	0.98	0.7873

Parameter	Association (slope dependent)	Lower 95 CI	Upper 95 CI	HR	*p*
Respiratory rate [/min]	0.5877	− 0.00857	1.1840	1.80	0.0534
Minute ventilation [L]	− 1.0156	− 1.6527	− 0.3784	0.36	0.0019
Tidal volume [ml/kg of ideal bodyweight]	− 2.7083	− 4.0486	− 1.3681	0.07	< 0.0001
Driving pressure [mbar]	1.9606	1.2518	2.6695	7.10	< 0.0001
Log_2_ of Horovitz index [mmHg]	− 8.3579	− 12.4653	− 4.2505	0.0002	< 0.0001
Log_2_ of Procalcitonin [ng/ml]	2.4995	1.6955	3.3034	12.18	< 0.0001
PEEP [mbar]	2.6039	1.8329	3.3750	13.52	< 0.0001
P_peak_ [mbar]	1.1877	0.7618	1.6137	3.28	< 0.0001
CRP (sqrt) [mg/l]	1.4467	0.8911	2.0023	4.25	< 0.0001
Leucocytes (sqrt) [GPt/L]	0.001461	− 2.6301	2.6331	1.00	0.9991

Data are association coefficient, 95% confidence interval (CI), Hazard ratio (HR), significance (p). *CRP* C-reactive protein; *GPt* gigaparticles; *L* litre; *Log*_*2*_ logarithm to base 2; *PEEP* positive end-expiratory pressure; *P*_*peak*_ peak airway pressure; *sqrt* square root

## Discussion

The main finding of this study is the strong association between the change in the slope of progression curves and patient survival.

This study examined the clinical characteristics of 274 patients with ARDS caused by primary pneumonia, one of the most common causes of ARDS [3, 4]. The application of the aforementioned selection criteria has resulted in the formation of a relatively homogeneous cohort, which differs from a typical ARDS cohort due to the high prevalence of severe ARDS and the high number of ECMO therapies performed. Due to the high severity of the disease and the resulting prolonged length of stay, a substantial amount of data was collected for the implementation of a statistical procedure that has been relatively underutilised in the context of ARDS to date: joint modelling. The use of joint models revealed that dynamic changes, as illustrated by alterations in the trajectory of the parameters, exert a markedly impact on mortality.

This finding has also implications for the clinical decision-making process. The decision-making process in the ICU is typically less influenced by the presence of risk factors for mortality at the time of admission than by the progression of the disease over time. Given the retrospective nature of the study and the narrow inclusion criteria, the findings pertain to the complete duration of the patients'ICU stay. Nevertheless, as illustrated in Figs. 2–4, there may be notable discrepancies between survivors and non-survivors during the initial days of ICU stay in this severely ill patient cohort. Accordingly, further, large-scale studies could concentrate on the course of events during the initial few days of hospitalisation. We are confident that a combination of sophisticated modelling with artificial intelligence, for instance, can enhance decision-making in routine clinical practice. The application of artificial intelligence in the field of diagnostics [18, 19], classification [20] or therapy control [21] has been previously documented in literature.

The PaO_2_/F_I_O_2_ ratio is a well-established risk factor for poor outcomes [22, 23]. Furthermore, it serves as a marker for severity classification in accordance with the Berlin definition of ARDS [2]. However, Fig. 3 demonstrates that the discrepancy in the PaO_2_/F_I_O_2_ ratio is not yet statistically significant. Consequently, no risk assessment could be conducted at this stage. This distinction can only be made when examining the subsequent progression of the disease, as the survival and non-survival curves diverge significantly. Our findings suggest that it may be more crucial to focus on the change (slope-dependent association) than on the absolute level of the value (current value association).

The negative associations of tidal volume with mortality for current values and progression-dependent values may seem surprising. One possible explanation could be the correlation between low tidal volume and poor compliance with poor outcomes. Notably, the effect shown is limited by the boundaries of lung-protective ventilation and even lower tidal volumes used during ECMO therapy. The proportion of patients receiving ECMO therapy at each day of stay is shown in Table e12. In general, therapeutic interventions were not considered as confounders in the present model, as the 24-h resolution does not adequately capture treatments that are significantly shorter in duration. The focus of the study is on the depiction of the clinical course, including therapeutic measures and complications. The effectiveness of a treatment is only reflected indirectly through the improvement of the respective parameter over time, which is then incorporated into the modelling: for example, prone positioning may contribute to improved oxygenation, and better oxygenation is associated with increased survival in our model. However, the model cannot determine whether the increased survival is attributable to the prone positioning itself or to the clinical improvement in general. It is likely that both aspects play an important role. A definitive conclusion regarding a causal relationship cannot be drawn due to the retrospective nature of the study. The associations only provide an idea of the correlations between the progression parameters.

The cohort analysed represents only a proportion of patients with ARDS. Nevertheless, discernible outcomes were evident for this specific subgroup. Further investigation should be conducted into all causes of ARDS. In particular, joint modelling could assist in the identification of phenotypes throughout the course of the disease, thereby facilitating a more individualised approach to therapy. A study was conducted by Bos et al. with the objective of identifying a subphenotype of Covid-19 related ARDS using longitudinal data [24]. The majority of other ARDS phenotypes have only been described using fixed time parameters [25, 26].

In addition, our study identified several other risk factors associated with adverse outcomes in ARDS patients, including advanced age or SARS-CoV-2 infection.

### Study limitations

Firstly, the retrospective design of our analysis carries the risk of bias and incomplete data, which may result in the overlooking of crucial contextual factors that influence patient outcomes. Secondly, the selection of patients based on ICD codes is susceptible to the potential for misclassification, whereby errors in coding may result in the exclusion or misrepresentation of patients. Thirdly, a considerable proportion of cases involved patients with confirmed diagnoses of SARS-CoV-2 infection. A number of studies have been conducted to examine the differences between typical ARDS and that observed in patients with SARS-CoV-2 infection, with a particular focus on the incidence of thromboembolic events and respiratory compliance [27, 28]. However, all of the patients in question had been diagnosed with viral pneumonia and met the inclusion criteria. Fourthly, the lengthy observation period encompasses alterations in medical practice, necessitating meticulous interpretation of the findings amidst evolving clinical paradigms. A further limitation of the study is that the proportion of spontaneous breathing was not analysed, which restricts the assessment of driving pressure. Ultimately, in order to apply the joint models, it was necessary to adjust for shifted data. This necessitated the utilisation of the logarithm to base 2 for the PaO_2_/F_I_O_2_ ratio and procalcitonin, in addition to the square root of CRP and leukocytes, for the model. While this allows for the utilisation of the model, it does not always reflect the practical realities of clinical practice, particularly when logarithmising to base 2. This is due to the fact that when utilising the Log2 function, an increase of one unit results in a doubling of the actual value. To illustrate, an increase from a PaO₂/F_I_O₂ ratio of 128 (2^7^) mmHg to 256 (2^8^) mmHg, and subsequently to 512 (2^9^) mmHg, can be observed. A more realistic increase, for example from 128 (2^7^) mmHg to 147 (2^7.2^) mmHg, would correspond to approximately only one fifth of the reported HR. These considerable fluctuations also account for the elevated hazard ratios, particularly in the case of PaO₂/F_I_O₂ and procalcitonin.

## Conclusion

This study demonstrated the importance of determining the clinical time course of parameters in ARDS patients. The increasing slopes of the curves of the respiratory and infectious parameters revealed an important and significant predictive value. The dynamics of routine parameters should therefore be prioritized for risk assessment and therapeutic management in ARDS patients.

## Supplementary Information

Additional file 1.

## Data Availability

Data is provided within the manuscript or supplementary information files.

## Abbreviations

ACE: Angiotensin-converting enzyme
At 2: Angiotensin II
ARDS: Acute respiratory distress syndrome
BMI: Body mass index
CCI: Charlson comorbidity index
CI: Confidence interval
CRP: C-reactive protein
CRRT: Continuous renal replacement therapy
DOACs: Direct oral anticoagulation
ECMO: Extracorporeal membrane oxygenation
FIO_2_: Inspiratory oxygen fraction
GCS: Glasgow Coma Scale
HR: Hazard ratio
ICU: Intensive care unit
LMWH: Low-molecular weight heparin
PaO_2_: Partial pressure of oxygen
PEEP: Positive end-expiratory pressure
Ppeak: Peak inspiratory pressure
SOFA: Sequential organ failure assessment
SOPs: Standard operating procedures
SPSS: Statistical package for the social sciences
STROBE: Strengthening the reporting of observational studies in epidemiology
UFH: Unfractionated heparin
