# Supplementary material for: Impact of ventilatory and laboratory parameter trajectories on short-term survival in acute respiratory distress syndrome patients: a retrospective study using joint models
Source: Eur J Med Res. 2025 May 20;30:406. doi: 10.1186/s40001-025-02650-z (PMC12090381; doi:10.1186/s40001-025-02650-z)
Supplement: Supplementary file 1 — Additional file 1. [file 40001_2025_2650_MOESM1_ESM.docx]

**Table e1: Additional descriptive statistics**

|  | Non-Survivor | range | Survivor | range | *p* |
| --- | --- | --- | --- | --- | --- |
| n | 136 |  | 138 |  |  |
| Male | 107 (78.7 %) |  | 93 (67.4 %) |  | *0.041* |
| Age [years] | 67 (59; 73) | 33-92 | 62 (55; 69) | 20-83 | *<0.001* |
| Body-Mass-Index [kg/m^2^] | 27.7 (25.0; 31.0) | 17.3-70.3 | 29.4 (25.4; 33.4) | 18.9-66.8 | *0.085* |
| COVID-19 | 93 (68.4 %) |  | 90 (65.2 %) |  | *0.609* |
| Viruspneumonia | 100 (73.5 %) |  | 96 (69.6 %) |  | *0.505* |
| Bacterial pneumonia | 9 (6.6 %) |  | 23 (16.7 %) |  | *0.014* |
| Pneumonia without proven pathogen | 27 (19.9 %) |  | 19 (13.8 %) |  | *0.198* |
| Direct transfer to our ICU from other hospital | 97 (71.3 %) |  | 98 (71.0 %) |  | *1.000* |
| External tracheostomy | 11 (8.1 %) |  | 11 (8.0 %) |  | *1.000* |
| External intubation | 110 (80.9 %) |  | 110 (79.7 %) |  | *0.880* |
| Invasive mechanical ventilation before admission to our ICU [days\| | 2 (0; 6) | 0-28 | 1 (0; 4) | 0-20 | *0.191* |
| Non-invasive mechanical ventilation before admission to our ICU [days\| | 1 (0;3) | 0-28 | 1 (0; 4) | 0-20 | *0.106* |
| Charlson comorbidity index | 4 (2; 6) | 0-11 | 3 (2; 5) | 0-11 | *0.008* |
| Arterial Hypertension | 93 (68.4 %) |  | 97 (70.3 %) |  | *0.794* |
| Cardiovascular disease | 37 (27.2 %) |  | 31 (22.5 %) |  | *0.403* |
| Neurovascular symptoms | 15 (11.0 %) |  | 12 (8.7 %) |  | *0.549* |
| Thrombembolic events in medical history | 11 (8.1 %) |  | 5 (3.6 %) |  | *0.130* |
| Chronic arrhythmias | 35 (25.7 %) |  | 25 (18.1 %) |  | *0.145* |
| COPD | 10 (7.4 %) |  | 11 (8.0 %) |  | *1.000* |
| Other pulmonary disease | 11 (8.1 %) |  | 8 (5.8 %) |  | *0.485* |
| Nicotine abuse | 16 (11.8 %) |  | 27 (19.6 %) |  | *0.096* |
| Diabetes mellitus | 51 (37.5 %) |  | 57 (41.3 %) |  | *0.539* |
| Previous organ or bone marrow transplantation | 4 (2.9 %) |  | 6 (4.3 %) |  | *0.749* |
| PaCO_2_ at admission [kPa] | 6.65 (5.78; 7.93) | 3.95-15.90 | 6.42 (5.64; 7.45) | 3.23-18.00 | *0.111* |
| SpO_2_ at admission [%] | 93 (90; 96) | 64-100 | 94 (91; 96) | 56-100 | *0.187* |
| Reintubation | 6 (4.4 %) |  | 11 (8.0 %) |  | *0.317* |
| Tracheostomy | 51 (37.5 %) |  | 65 (47.1 %) |  | *0.114* |
| Red Cell Transfusion | 121 (89.0 %) |  | 79 (57.2 %) |  | *<0.001* |
| Red Cell Transfusion [n] | 8 (3; 15) | 1-62 | 6 (2; 16) | 1-42 | *0.337* |
| Corticosteroid | 126 (92.6 %) |  | 96 (69.6 %) |  | *<0.001* |
| Dexamethasone | 64 (47.1 %) |  | 63 (45.7 %) |  | *0.904* |
| Hydrocortisone | 86 (63.2 %) |  | 28 (20.3 %) |  | *<0.001* |
| Other Corticosteroids | 41 (30.1 %) |  | 24 (17.4 %) |  | *0.016* |
| Antibiotics | 133 (97.8 %) |  | 132 (95.7 %) |  | *0.501* |
| Antimycotics | 29 (21.3 %) |  | 19 (13.8 %) |  | *0.113* |
| Coagulase-negative staphylococci in the blood culture | 31 (22.8 %) |  | 26 (18.8 %) |  | *0.459* |
| Streptococci in the blood culture | 3 (2.2 %) |  | 2 (1.4 5) |  | *0.683* |
| Enterococci in the blood culture | 17 (12.5 %) |  | 8 (5.8 %) |  | *0.061* |
| Staphylococcus aureus in the blood culture | 9 (6.6 %) |  | 2 (1.4 5) |  | *0.034* |
| Klebsiella in the blood culture | 13 (9.6 %) |  | 6 (4.3 %) |  | *0.101* |
| Non-fermenters in the blood culture | 4 (2.9 %) |  | 0 (0%) |  | *0.590* |
| Escherichia coli in the blood culture | 4 (2.9 %) |  | 10 (7.2 %) |  | *0.168* |
| Proteus in the blood culture | 0 (0 %) |  | 0 (0%) |  |  |
| Filamentous fungi in the blood culture | 0 (0 %) |  | 0 (0%) |  |  |
| Yeasts in the blood culture | 10 (7.4 %) |  | 4 (2.9 %) |  | *0.107* |
| Interleukin 6 maximum value [pg/mL] | 601 (250.5; 2044) | 15.9-792732 | 159 (85; 398) | 8.6-21728 | *<0.001* |
| Prothrombin fragment F1+2 maximum value [pmol/l] | 544 (345; 940) | 73-4948 | 395 (233; 781) | 98-4948 | *0.075* |
| Platelets maximum value [GPt/L] | 245 (164; 333) | 48-617 | 353 (288; 484) | 74-989 | *<0.001* |
| Platelets minimum value [GPt/L] | 71 (34; 118) | 1-414 | 144 (84; 209) | 4-469 | *<0.001* |
| DVT | 36 (26.5 %) |  | 26 (18.8 %) |  | *0.150* |
| Catheter associated thrombosis | 5 (3.7 %) |  | 7 (5.1 %) |  | *0.769* |
| PE | 33 (24.3 %) |  | 27 (19.6 %) |  | *0.382* |
| Pneumothorax | 23 (17.0 %) |  | 22 (15.9 %) |  | *0.871* |
| Lung emphysema | 6 (10.7 %) |  | 2 (3.7 %) |  | *0.271* |
| Mediastinal emphysema | 9 (15.5 %) |  | 5 (9.1 %) |  | *0.395* |
| Subcutaneous emphysema | 14 (24.6 %) |  | 10 (18.9 %) |  | *0.498* |
| Pleural effusion | 65 (48.1 %) |  | 51 (37.0 %) |  | *0.067* |

Table e1: Data are median (Interquartile range) or n (%). COPD: Chronic obstructive pulmonary disease; DVT: Deep vein thrombosis; ICU: Intensive care unit; PaCO_2_: partial pressure of carbon dioxide; PE: Pulmonary embolism; SpO_2_: Oxygen saturation;

**Table e2: Respiratory rate**

|  | Non-Survivor | | | Survivor | | |
| --- | --- | --- | --- | --- | --- | --- |
| Day of stay | Median | Lower limit of 95% CI of the median | Upper limit of 95% CI of the median | Median | Lower limit of 95% CI of the median | Upper limit of 95% CI of the median |
| 1 | 23.27 | 21.79 | 25.67 | 23.57 | 22.69 | 24.77 |
| 2 | 22.79 | 21.33 | 26.81 | 24.38 | 23.32 | 25.11 |
| 3 | 24.43 | 22.24 | 25.88 | 24.27 | 22.69 | 25.33 |
| 4 | 24.72 | 23.25 | 25.55 | 23.94 | 22.32 | 25.19 |
| 5 | 24.77 | 23.80 | 25.83 | 23.49 | 21.81 | 24.88 |
| 6 | 24.75 | 23.97 | 26.21 | 24.06 | 22.43 | 25.50 |
| 7 | 25.16 | 23.72 | 26.36 | 23.92 | 22.71 | 25.42 |
| 8 | 25.50 | 24.00 | 27.28 | 24.37 | 22.96 | 26.09 |
| 9 | 26.15 | 25.41 | 27.28 | 25.20 | 24.28 | 26.42 |
| 10 | 26.48 | 25.50 | 28.07 | 24.25 | 23.07 | 26.16 |
| 11 | 25.96 | 24.52 | 28.57 | 25.27 | 23.97 | 26.44 |
| 12 | 26.36 | 24.65 | 28.92 | 24.68 | 23.68 | 26.42 |
| 13 | 26.58 | 25.64 | 29.08 | 25.01 | 24.04 | 26.92 |
| 14 | 27.40 | 25.79 | 29.68 | 24.89 | 23.44 | 27.44 |
| 15 | 28.90 | 26.13 | 30.21 | 25.84 | 23.71 | 27.33 |
| 16 | 28.85 | 27.16 | 30.26 | 26.26 | 21.69 | 28.92 |
| 17 | 28.79 | 24.54 | 30.56 | 25.50 | 23.25 | 27.83 |
| 18 | 28.00 | 26.26 | 30.08 | 26.04 | 24.78 | 28.60 |
| 19 | 28.96 | 26.00 | 30.73 | 26.41 | 24.94 | 28.77 |
| 20 | 27.50 | 25.48 | 30.00 | 27.00 | 25.54 | 29.19 |
| 21 | 28.13 | 26.00 | 30.33 | 27.61 | 23.43 | 29.78 |
| 22 | 28.73 | 26.00 | 30.00 | 28.63 | 26.25 | 31.06 |
| 23 | 27.70 | 21.28 | 30.00 | 25.91 | 24.44 | 30.35 |
| 24 | 26.24 | 22.11 | 27.75 | 28.21 | 25.26 | 29.80 |
| 25 | 24.00 | 20.96 | 28.08 | 30.04 | 25.96 | 34.23 |
| 26 | 25.83 | 15.72 | 32.89 | 29.64 | 27.31 | 32.52 |
| 27 | 27.50 | 25.38 | 30.21 | 30.47 | 27.42 | 33.50 |
| 28 | 24.28 | 21.81 | 31.40 | 29.85 | 27.62 | 31.80 |
| 29 | 26.92 | 21.59 | 31.67 | 30.18 | 27.20 | 34.54 |
| 30 | 29.13 | 25.15 | 30.58 | 29.70 | 26.54 | 33.84 |
| 31 | 28.39 | 25.00 | 31.07 | 27.00 | 24.17 | 34.82 |
| 32 | 29.69 | 26.50 | 31.42 | 27.12 | 23.65 | 30.90 |
| 33 | 28.07 | 25.16 | 35.46 | 27.43 | 21.95 | 31.78 |
| 34 | 29.02 | 25.04 | 30.48 | 28.89 | 26.88 | 31.00 |
| 35 | 28.13 | 24.44 | 33.44 | 31.38 | 29.56 | 33.77 |
| 36 | 27.21 | 26.76 | 30.00 | 26.96 | 23.28 | 33.21 |
| 37 | 28.32 | 26.71 | 29.67 | 28.34 | 22.28 | 36.93 |
| 38 | 28.64 | 25.04 | 33.89 | 27.24 | 25.11 | 32.96 |
| 39 | 30.46 | 24.76 | 34.00 | 25.56 | 21.83 | 29.50 |
| 40 | 32.81 | 25.73 | 36.07 | 27.21 | 22.28 | 32.21 |
| 41 | 32.01 | 23.53 | 37.73 | 25.44 | 24.61 | 33.35 |
| 42 | 28.29 | 22.15 | 34.46 | 27.12 | 23.92 | 32.43 |
| 43 | 28.36 | 23.52 | 40.12 | 27.68 | 21.59 | 36.00 |
| 44 | 29.21 | 26.65 | 38.28 | 31.50 | 23.48 | 36.00 |
| 45 | 29.26 | 29.13 | 37.54 | 32.62 | 23.96 | 34.44 |
| 46 | 28.81 | 26.33 | 35.60 | 30.00 | 25.46 | 31.80 |
| 47 | 35.29 | 30.41 | 40.17 | 28.50 | 25.46 | 35.21 |
| 48 | 35.28 | 26.41 | 44.15 | 34.59 | 28.76 | 36.77 |
| 49 | 36.42 | 32.59 | 40.24 | 25.08 | 24.04 | 37.52 |
| 50 | 31.33 | 25.92 | 36.73 | 25.50 | 20.83 | 47.52 |
| 51 | 25.64 |  |  | 22.96 | 22.77 | 39.29 |
| 52 | 27.27 |  |  | 26.54 | 25.04 | 43.36 |
| 53 | 30.54 |  |  | 28.21 | 24.19 | 40.42 |
| 54 | 30.57 |  |  | 34.52 | 28.76 | 38.54 |
| 55 | 28.85 |  |  | 34.73 | 31.96 | 37.50 |
| 56 | 29.27 |  |  | 33.90 | 29.80 | 38.00 |
| 57 | 29.37 |  |  | 33.46 | 28.70 | 38.21 |
| 58 | 31.41 |  |  | 33.10 | 27.58 | 38.62 |
| 59 | 34.97 |  |  | 33.46 | 29.67 | 37.25 |
| 60 | 33.52 |  |  | 33.02 | 28.71 | 37.33 |
| 61 |  |  |  | 32.93 | 27.64 | 38.21 |
| 62 |  |  |  | 36.17 | 31.19 | 41.15 |
| 63 |  |  |  | 34.96 | 29.84 | 40.08 |
| 64 |  |  |  | 34.39 | 29.05 | 39.72 |
| 65 |  |  |  | 33.70 | 25.77 | 41.63 |
| 66 |  |  |  | 33.69 | 25.25 | 42.13 |
| 67 |  |  |  | 35.08 | 27.81 | 42.35 |
| 68 |  |  |  | 33.86 | 26.50 | 41.21 |
| 69 |  |  |  | 27.92 | 25.71 | 30.13 |
| 70 |  |  |  | 34.36 | 27.58 | 41.13 |
| 71 |  |  |  | 31.18 | 24.10 | 38.25 |
| 72 |  |  |  | 33.14 | 27.52 | 38.75 |
| 73 |  |  |  | 33.66 | 28.12 | 39.20 |
| 74 |  |  |  | 32.72 | 27.76 | 37.68 |
| 75 |  |  |  | 30.93 | 23.64 | 38.21 |
| 76 |  |  |  | 30.49 | 23.46 | 37.52 |
| 77 |  |  |  | 31.56 | 25.74 | 37.38 |
| 78 |  |  |  | 31.69 | 26.00 | 37.38 |
| 79 |  |  |  | 30.82 | 24.64 | 37.00 |
| 80 |  |  |  | 28.26 | 23.64 | 32.88 |
| 81 |  |  |  | 30.66 | 23.78 | 37.54 |
| 82 |  |  |  | 30.01 | 23.56 | 36.46 |
| 83 |  |  |  | 31.13 | 25.47 | 36.78 |
| 84 |  |  |  | 26.80 |  |  |
| 85 |  |  |  | 22.57 |  |  |
| 86 |  |  |  | 25.13 |  |  |
| 87 |  |  |  | 27.79 |  |  |
| 88 |  |  |  | 30.53 |  |  |

Table e2: Respiratory rate [1/min]; Data are median, its lower and its upper limit of 95% confidence interval (CI) of daily mean values of non-survivors and survivors at each day of stay

**Table e3: Positive end-expiratory pressure**

|  | Non-Survivor | | | Survivor | | |
| --- | --- | --- | --- | --- | --- | --- |
| Day of stay | Median | Lower limit of 95% CI of the median | Upper limit of 95% CI of the median | Median | Lower limit of 95% CI of the median | Upper limit of 95% CI of the median |
| 1 | 13.12 | 12.46 | 13.85 | 12.65 | 12.07 | 13.58 |
| 2 | 13.00 | 12.12 | 13.83 | 12.27 | 12.00 | 12.85 |
| 3 | 12.50 | 12.00 | 13.17 | 12.00 | 11.66 | 12.32 |
| 4 | 12.00 | 11.96 | 12.92 | 11.66 | 10.20 | 12.00 |
| 5 | 12.00 | 11.83 | 12.67 | 10.74 | 10.00 | 11.69 |
| 6 | 12.00 | 11.79 | 12.67 | 10.08 | 9.93 | 11.52 |
| 7 | 12.07 | 11.63 | 12.58 | 10.00 | 9.68 | 11.31 |
| 8 | 12.00 | 11.68 | 12.64 | 9.69 | 8.67 | 10.32 |
| 9 | 12.42 | 12.00 | 13.00 | 10.00 | 8.60 | 10.96 |
| 10 | 12.08 | 11.85 | 13.00 | 10.00 | 9.44 | 10.65 |
| 11 | 12.29 | 12.00 | 13.00 | 9.70 | 8.67 | 10.44 |
| 12 | 12.00 | 11.82 | 12.90 | 10.00 | 9.83 | 10.48 |
| 13 | 12.00 | 11.48 | 13.06 | 10.00 | 9.83 | 10.96 |
| 14 | 12.00 | 11.08 | 13.00 | 10.00 | 9.96 | 10.79 |
| 15 | 12.16 | 11.90 | 13.00 | 10.00 | 10.00 | 10.67 |
| 16 | 12.80 | 11.83 | 13.08 | 9.92 | 8.43 | 10.00 |
| 17 | 12.54 | 12.00 | 13.52 | 9.52 | 8.00 | 10.00 |
| 18 | 12.62 | 12.00 | 13.46 | 9.08 | 8.00 | 10.00 |
| 19 | 12.54 | 12.00 | 13.92 | 8.13 | 8.00 | 10.00 |
| 20 | 12.35 | 11.96 | 13.96 | 8.00 | 7.96 | 10.52 |
| 21 | 12.00 | 11.77 | 14.00 | 8.00 | 8.00 | 11.23 |
| 22 | 12.46 | 11.67 | 13.80 | 8.00 | 8.00 | 10.08 |
| 23 | 12.06 | 10.17 | 13.78 | 8.00 | 8.00 | 11.08 |
| 24 | 10.92 | 10.00 | 14.00 | 8.27 | 8.00 | 9.65 |
| 25 | 10.00 | 10.00 | 15.00 | 8.00 | 8.00 | 9.75 |
| 26 | 10.17 | 10.00 | 12.00 | 8.00 | 8.00 | 10.00 |
| 27 | 10.39 | 10.00 | 11.96 | 8.00 | 8.00 | 9.77 |
| 28 | 10.24 | 10.00 | 13.81 | 8.00 | 8.00 | 10.00 |
| 29 | 10.48 | 10.00 | 12.00 | 8.20 | 8.00 | 9.80 |
| 30 | 12.00 | 12.00 | 13.75 | 8.99 | 8.00 | 9.76 |
| 31 | 11.77 | 10.00 | 12.00 | 8.63 | 8.00 | 10.00 |
| 32 | 11.68 | 9.21 | 12.00 | 8.00 | 7.96 | 9.60 |
| 33 | 11.76 | 10.00 | 12.00 | 8.00 | 8.00 | 11.07 |
| 34 | 11.37 | 10.21 | 12.00 | 8.00 | 8.00 | 11.59 |
| 35 | 10.00 | 10.00 | 13.83 | 7.92 | 6.77 | 9.73 |
| 36 | 10.12 | 10.00 | 13.48 | 8.00 | 7.00 | 10.08 |
| 37 | 10.50 | 10.00 | 13.36 | 8.08 | 6.17 | 8.56 |
| 38 | 12.14 | 10.00 | 13.42 | 7.98 | 6.13 | 10.19 |
| 39 | 11.31 | 10.00 | 13.76 | 8.06 | 6.21 | 11.07 |
| 40 | 10.94 | 8.00 | 12.03 | 8.00 | 6.27 | 10.42 |
| 41 | 10.36 | 8.04 | 12.08 | 8.00 | 7.26 | 11.52 |
| 42 | 9.74 | 8.00 | 10.00 | 8.07 | 7.90 | 10.08 |
| 43 | 8.00 | 7.79 | 10.00 | 8.20 | 8.00 | 11.92 |
| 44 | 8.35 | 8.08 | 10.00 | 8.05 | 8.00 | 12.04 |
| 45 | 9.00 | 8.37 | 10.00 | 8.40 | 8.00 | 12.00 |
| 46 | 10.00 | 8.00 | 10.20 | 8.67 | 7.04 | 10.32 |
| 47 | 9.00 | 8.00 | 10.00 | 8.02 | 8.00 | 9.17 |
| 48 | 8.69 | 8.00 | 9.38 | 8.38 | 8.00 | 10.08 |
| 49 | 8.70 | 8.00 | 9.40 | 8.00 | 6.64 | 10.12 |
| 50 | 9.20 | 9.12 | 9.27 | 8.00 | 7.56 | 9.96 |
| 51 | 9.83 |  |  | 8.00 | 6.00 | 10.00 |
| 52 | 8.00 |  |  | 8.00 | 6.00 | 8.88 |
| 53 | 8.04 |  |  | 8.00 | 6.05 | 8.37 |
| 54 | 8.13 |  |  | 8.00 |  |  |
| 55 | 11.04 |  |  | 8.00 |  |  |
| 56 | 11.62 |  |  | 8.00 |  |  |
| 57 | 12.41 |  |  | 8.00 |  |  |
| 58 | 12.00 |  |  | 8.00 |  |  |
| 59 | 12.00 |  |  | 8.00 |  |  |
| 60 | 12.60 |  |  | 8.00 |  |  |
| 61 |  |  |  | 7.96 | 7.91 | 8.00 |
| 62 |  |  |  | 7.95 | 7.90 | 8.00 |
| 63 |  |  |  | 8.00 |  |  |
| 64 |  |  |  | 7.98 | 7.95 | 8.00 |
| 65 |  |  |  | 7.68 | 7.36 | 8.00 |
| 66 |  |  |  | 7.72 | 7.43 | 8.00 |
| 67 |  |  |  | 7.86 | 7.72 | 8.00 |
| 68 |  |  |  | 9.34 | 8.00 | 10.68 |
| 69 |  |  |  | 9.75 | 8.00 | 11.50 |
| 70 |  |  |  | 9.50 | 8.00 | 11.00 |
| 71 |  |  |  | 9.19 | 8.00 | 10.38 |
| 72 |  |  |  | 9.52 | 8.00 | 11.04 |
| 73 |  |  |  | 8.71 | 8.00 | 9.42 |
| 74 |  |  |  | 8.56 | 7.76 | 9.36 |
| 75 |  |  |  | 8.63 | 7.25 | 10.00 |
| 76 |  |  |  | 8.75 | 7.50 | 10.00 |
| 77 |  |  |  | 8.79 | 7.57 | 10.00 |
| 78 |  |  |  | 8.71 | 7.42 | 10.00 |
| 79 |  |  |  | 8.98 | 7.96 | 10.00 |
| 80 |  |  |  | 9.09 | 8.18 | 10.00 |
| 81 |  |  |  | 9.03 | 8.06 | 10.00 |
| 82 |  |  |  | 9.00 | 8.00 | 10.00 |
| 83 |  |  |  | 9.00 | 8.00 | 10.00 |
| 84 |  |  |  | 8.00 |  |  |
| 85 |  |  |  | 8.00 |  |  |
| 86 |  |  |  | 8.75 |  |  |
| 87 |  |  |  | 10.50 |  |  |
| 88 |  |  |  | 8.00 |  |  |

Table e3: Positive end-expiratory pressure [mbar]; Data are median, its lower and its upper limit of 95% confidence interval (CI) of daily mean values of non-survivors and survivors at each day of stay

**Table e4: Peak airway pressure**

|  | Non-Survivor | | | Survivor | | |
| --- | --- | --- | --- | --- | --- | --- |
| Day of stay | Median | Lower limit of 95% CI of the median | Upper limit of 95% CI of the median | Median | Lower limit of 95% CI of the median | Upper limit of 95% CI of the median |
| 1 | 28.16 | 26.60 | 28.74 | 26.59 | 26.09 | 27.42 |
| 2 | 27.12 | 26.25 | 28.08 | 25.48 | 25.04 | 26.38 |
| 3 | 26.92 | 26.16 | 27.72 | 24.91 | 24.08 | 25.75 |
| 4 | 26.72 | 25.88 | 27.48 | 24.04 | 22.81 | 24.79 |
| 5 | 26.83 | 26.32 | 27.67 | 23.41 | 22.17 | 24.04 |
| 6 | 27.16 | 26.11 | 28.07 | 22.60 | 21.50 | 24.00 |
| 7 | 27.00 | 25.92 | 28.11 | 21.68 | 20.05 | 23.62 |
| 8 | 26.46 | 25.52 | 27.81 | 22.14 | 18.80 | 24.17 |
| 9 | 27.21 | 26.17 | 27.76 | 22.48 | 19.71 | 24.38 |
| 10 | 27.67 | 26.96 | 28.38 | 22.41 | 20.83 | 24.96 |
| 11 | 27.86 | 26.44 | 29.19 | 22.00 | 20.67 | 23.91 |
| 12 | 28.00 | 26.46 | 29.07 | 22.60 | 21.40 | 24.78 |
| 13 | 27.98 | 27.12 | 29.54 | 24.64 | 22.08 | 25.56 |
| 14 | 28.76 | 27.08 | 30.50 | 23.64 | 22.14 | 25.04 |
| 15 | 29.19 | 27.18 | 30.21 | 23.50 | 21.88 | 25.00 |
| 16 | 29.04 | 28.38 | 29.92 | 23.08 | 20.83 | 24.83 |
| 17 | 29.33 | 27.85 | 30.88 | 22.40 | 20.13 | 25.20 |
| 18 | 29.79 | 27.75 | 31.18 | 22.94 | 18.92 | 25.38 |
| 19 | 29.62 | 28.35 | 30.75 | 23.60 | 20.96 | 25.31 |
| 20 | 30.12 | 27.83 | 31.28 | 23.60 | 21.43 | 26.27 |
| 21 | 29.53 | 28.17 | 30.26 | 23.74 | 21.46 | 25.80 |
| 22 | 30.00 | 28.17 | 31.83 | 23.59 | 21.85 | 25.46 |
| 23 | 30.32 | 27.44 | 31.80 | 23.32 | 20.91 | 26.15 |
| 24 | 27.85 | 26.68 | 31.33 | 24.10 | 22.65 | 26.38 |
| 25 | 26.96 | 25.13 | 31.94 | 24.63 | 21.78 | 25.79 |
| 26 | 27.98 | 25.25 | 32.14 | 24.07 | 21.48 | 26.20 |
| 27 | 27.55 | 25.73 | 31.08 | 21.82 | 20.75 | 24.71 |
| 28 | 27.39 | 24.36 | 34.06 | 22.79 | 20.32 | 24.05 |
| 29 | 28.79 | 21.04 | 32.80 | 24.38 | 20.19 | 25.42 |
| 30 | 28.68 | 22.58 | 34.56 | 24.36 | 22.25 | 26.68 |
| 31 | 28.27 | 23.08 | 36.17 | 22.25 | 19.79 | 29.00 |
| 32 | 27.69 | 24.24 | 38.96 | 23.37 | 19.88 | 27.64 |
| 33 | 29.88 | 27.04 | 32.59 | 22.17 | 20.37 | 24.78 |
| 34 | 27.97 | 26.91 | 29.74 | 23.21 | 18.67 | 28.89 |
| 35 | 28.04 | 27.04 | 31.83 | 23.50 | 19.39 | 27.73 |
| 36 | 27.88 | 27.46 | 31.60 | 25.09 | 18.17 | 30.73 |
| 37 | 29.92 | 27.54 | 32.12 | 25.80 | 17.81 | 28.52 |
| 38 | 31.00 | 27.21 | 33.04 | 26.50 | 17.93 | 29.19 |
| 39 | 30.09 | 25.23 | 33.07 | 26.42 | 18.50 | 28.76 |
| 40 | 26.64 | 24.50 | 34.39 | 26.18 | 19.68 | 30.42 |
| 41 | 26.79 | 24.96 | 35.64 | 26.29 | 21.11 | 31.59 |
| 42 | 26.78 | 24.50 | 30.54 | 26.89 | 22.08 | 30.76 |
| 43 | 23.76 | 20.70 | 30.42 | 28.72 | 20.14 | 35.08 |
| 44 | 23.67 | 23.04 | 31.48 | 28.21 | 20.22 | 35.12 |
| 45 | 23.11 | 22.96 | 29.96 | 26.72 | 22.79 | 34.50 |
| 46 | 23.15 | 22.46 | 31.76 | 26.98 | 26.20 | 31.24 |
| 47 | 28.28 | 24.22 | 32.33 | 25.27 | 22.92 | 28.63 |
| 48 | 27.27 | 23.15 | 31.38 | 26.14 | 25.90 | 35.15 |
| 49 | 27.14 | 22.67 | 31.60 | 28.63 | 25.04 | 32.76 |
| 50 | 29.63 | 22.60 | 36.65 | 27.08 | 23.46 | 33.50 |
| 51 | 22.14 |  |  | 28.25 | 26.00 | 34.13 |
| 52 | 21.65 |  |  | 25.46 | 24.13 | 35.56 |
| 53 | 20.68 |  |  | 24.63 | 16.90 | 26.95 |
| 54 | 21.92 |  |  | 29.29 | 23.72 | 29.38 |
| 55 | 27.31 |  |  | 25.58 | 20.78 | 30.38 |
| 56 | 27.92 |  |  | 24.46 | 18.55 | 30.36 |
| 57 | 29.42 |  |  | 26.83 | 22.70 | 30.96 |
| 58 | 29.93 |  |  | 26.14 | 21.95 | 30.32 |
| 59 | 29.93 |  |  | 24.52 | 18.78 | 30.25 |
| 60 | 31.60 |  |  | 22.92 | 17.88 | 27.96 |
| 61 |  |  |  | 22.41 | 16.73 | 28.08 |
| 62 |  |  |  | 22.74 | 17.48 | 28.00 |
| 63 |  |  |  | 22.18 | 17.32 | 27.04 |
| 64 |  |  |  | 22.31 | 17.73 | 26.88 |
| 65 |  |  |  | 23.36 | 19.64 | 27.08 |
| 66 |  |  |  | 22.16 | 16.90 | 27.42 |
| 67 |  |  |  | 26.86 | 25.72 | 28.00 |
| 68 |  |  |  | 27.04 | 26.25 | 27.82 |
| 69 |  |  |  | 24.09 | 21.21 | 26.96 |
| 70 |  |  |  | 26.25 | 25.50 | 27.00 |
| 71 |  |  |  | 26.24 | 25.83 | 26.64 |
| 72 |  |  |  | 28.26 | 25.96 | 30.56 |
| 73 |  |  |  | 25.63 | 25.17 | 26.08 |
| 74 |  |  |  | 25.04 | 23.40 | 26.68 |
| 75 |  |  |  | 24.68 | 22.32 | 27.04 |
| 76 |  |  |  | 24.79 | 22.65 | 26.92 |
| 77 |  |  |  | 23.80 | 21.09 | 26.50 |
| 78 |  |  |  | 24.67 | 23.25 | 26.08 |
| 79 |  |  |  | 26.42 | 25.76 | 27.08 |
| 80 |  |  |  | 23.96 | 22.15 | 25.77 |
| 81 |  |  |  | 24.44 | 24.38 | 24.50 |
| 82 |  |  |  | 25.11 | 23.88 | 26.33 |
| 83 |  |  |  | 26.07 | 24.57 | 27.56 |
| 84 |  |  |  | 26.00 |  |  |
| 85 |  |  |  | 23.71 |  |  |
| 86 |  |  |  | 28.25 |  |  |
| 87 |  |  |  | 28.29 |  |  |
| 88 |  |  |  | 17.33 |  |  |

Table e4: Peak airway pressure [mbar]; Data are median, its lower and its upper limit of 95% confidence interval (CI) of daily mean values of non-survivors and survivors at each day of stay

**Table e5: Minute ventilation**

|  | Non-Survivor | | | Survivor | | |
| --- | --- | --- | --- | --- | --- | --- |
| Day of stay | Median | Lower limit of 95% CI of the median | Upper limit of 95% CI of the median | Median | Lower limit of 95% CI of the median | Upper limit of 95% CI of the median |
| 1 | 9.660 | 8.850 | 10.490 | 9.890 | 9.260 | 10.640 |
| 2 | 9.560 | 9.210 | 10.210 | 9.950 | 9.440 | 10.730 |
| 3 | 9.745 | 8.770 | 10.360 | 10.320 | 9.580 | 10.910 |
| 4 | 9.880 | 9.310 | 11.210 | 10.600 | 9.910 | 11.090 |
| 5 | 10.620 | 9.720 | 11.490 | 10.480 | 10.020 | 11.130 |
| 6 | 10.830 | 10.310 | 11.580 | 10.650 | 9.630 | 11.290 |
| 7 | 11.350 | 9.760 | 11.970 | 11.210 | 10.360 | 11.840 |
| 8 | 10.500 | 9.700 | 11.600 | 11.430 | 10.490 | 12.000 |
| 9 | 11.020 | 9.360 | 12.170 | 11.845 | 10.910 | 12.620 |
| 10 | 11.060 | 9.830 | 12.360 | 12.060 | 11.090 | 12.880 |
| 11 | 10.825 | 9.090 | 11.510 | 11.420 | 10.710 | 12.690 |
| 12 | 10.630 | 9.080 | 11.930 | 12.180 | 10.310 | 13.340 |
| 13 | 10.770 | 8.910 | 12.000 | 11.440 | 10.400 | 12.720 |
| 14 | 11.025 | 9.610 | 12.300 | 11.670 | 10.330 | 12.440 |
| 15 | 11.250 | 9.880 | 12.410 | 12.030 | 10.750 | 12.760 |
| 16 | 11.670 | 10.090 | 12.870 | 11.240 | 9.600 | 12.010 |
| 17 | 11.530 | 9.720 | 12.210 | 11.150 | 10.480 | 12.340 |
| 18 | 11.190 | 9.360 | 12.670 | 11.320 | 10.900 | 12.180 |
| 19 | 10.480 | 9.440 | 12.220 | 11.220 | 9.930 | 12.930 |
| 20 | 10.510 | 7.380 | 11.580 | 12.030 | 10.410 | 13.100 |
| 21 | 9.670 | 5.450 | 10.720 | 11.360 | 10.280 | 12.200 |
| 22 | 10.600 | 5.620 | 12.010 | 11.880 | 10.820 | 12.840 |
| 23 | 9.750 | 3.960 | 11.740 | 10.890 | 10.240 | 13.390 |
| 24 | 10.550 | 2.530 | 12.630 | 11.225 | 10.010 | 13.760 |
| 25 | 9.260 | 2.630 | 11.400 | 11.330 | 9.490 | 13.200 |
| 26 | 10.030 | 1.530 | 12.370 | 11.110 | 10.460 | 13.940 |
| 27 | 8.680 | 2.380 | 12.250 | 12.510 | 9.970 | 14.320 |
| 28 | 9.580 | 2.760 | 13.520 | 11.735 | 11.060 | 13.830 |
| 29 | 10.370 | 2.820 | 12.160 | 12.635 | 10.900 | 13.500 |
| 30 | 9.885 | 2.700 | 13.970 | 12.805 | 11.710 | 13.840 |
| 31 | 9.890 | 2.720 | 13.130 | 12.600 | 10.430 | 14.500 |
| 32 | 10.160 | 2.200 | 15.600 | 12.010 | 10.330 | 13.540 |
| 33 | 10.640 | 3.100 | 16.870 | 12.410 | 11.330 | 13.370 |
| 34 | 10.790 | 3.780 | 13.490 | 12.765 | 10.580 | 13.860 |
| 35 | 12.960 | 9.610 | 16.780 | 11.530 | 10.490 | 14.160 |
| 36 | 10.920 | 9.740 | 15.940 | 11.050 | 10.280 | 13.450 |
| 37 | 11.400 | 9.190 | 13.690 | 12.030 | 8.570 | 14.240 |
| 38 | 10.700 | 9.170 | 12.580 | 11.345 | 9.300 | 14.670 |
| 39 | 12.020 | 9.610 | 12.800 | 11.290 | 8.560 | 15.740 |
| 40 | 11.430 | 9.080 | 14.730 | 11.180 | 9.350 | 16.770 |
| 41 | 10.720 | 8.510 | 13.790 | 14.600 | 11.390 | 17.820 |
| 42 | 11.480 | 9.070 | 16.050 | 10.130 | 9.200 | 18.910 |
| 43 | 12.540 | 9.910 | 16.250 | 11.640 | 8.250 | 21.080 |
| 44 | 12.160 | 10.910 | 16.260 | 13.650 | 8.960 | 22.120 |
| 45 | 11.990 | 11.730 | 15.890 | 13.730 | 9.400 | 22.600 |
| 46 | 11.610 | 9.020 | 14.020 | 15.755 | 10.000 | 20.270 |
| 47 | 11.035 | 9.920 | 12.150 | 15.390 | 11.310 | 17.860 |
| 48 | 12.240 | 10.930 | 13.550 | 15.500 | 14.390 | 19.560 |
| 49 | 11.110 | 10.020 | 12.200 | 13.860 | 12.760 | 18.180 |
| 50 | 9.575 | 9.050 | 10.100 | 14.080 | 12.150 | 22.670 |
| 51 | 9.400 |  |  | 14.490 | 10.940 | 20.550 |
| 52 | 10.270 |  |  | 16.490 | 11.920 | 24.500 |
| 53 | 11.000 |  |  | 14.990 | 13.720 | 19.920 |
| 54 | 11.140 |  |  | 14.160 | 13.490 | 18.940 |
| 55 | 10.240 |  |  | 14.220 | 13.640 | 14.800 |
| 56 | 10.330 |  |  | 13.940 | 12.870 | 15.010 |
| 57 | 9.460 |  |  | 14.350 | 13.870 | 14.830 |
| 58 | 10.600 |  |  | 14.430 | 14.020 | 14.840 |
| 59 | 9.880 |  |  | 14.595 | 13.790 | 15.400 |
| 60 | 9.290 |  |  | 14.320 | 12.790 | 15.850 |
| 61 |  |  |  | 13.045 | 12.530 | 13.560 |
| 62 |  |  |  | 14.430 | 12.440 | 16.420 |
| 63 |  |  |  | 14.560 | 12.290 | 16.830 |
| 64 |  |  |  | 15.105 | 12.810 | 17.400 |
| 65 |  |  |  | 14.120 | 11.720 | 16.520 |
| 66 |  |  |  | 14.525 | 11.830 | 17.220 |
| 67 |  |  |  | 15.865 | 12.020 | 19.710 |
| 68 |  |  |  | 14.430 | 10.230 | 18.630 |
| 69 |  |  |  | 13.245 | 10.720 | 15.770 |
| 70 |  |  |  | 15.585 | 12.270 | 18.900 |
| 71 |  |  |  | 14.285 | 11.200 | 17.370 |
| 72 |  |  |  | 14.360 | 11.440 | 17.280 |
| 73 |  |  |  | 15.000 | 12.050 | 17.950 |
| 74 |  |  |  | 14.330 | 11.630 | 17.030 |
| 75 |  |  |  | 14.140 | 10.400 | 17.880 |
| 76 |  |  |  | 13.840 | 10.360 | 17.320 |
| 77 |  |  |  | 13.905 | 11.130 | 16.680 |
| 78 |  |  |  | 14.300 | 11.300 | 17.300 |
| 79 |  |  |  | 14.395 | 11.040 | 17.750 |
| 80 |  |  |  | 14.430 | 12.110 | 16.750 |
| 81 |  |  |  | 14.785 | 12.140 | 17.430 |
| 82 |  |  |  | 15.070 | 12.000 | 18.140 |
| 83 |  |  |  | 14.825 | 13.600 | 16.050 |
| 84 |  |  |  | 13.540 |  |  |
| 85 |  |  |  | 10.030 |  |  |
| 86 |  |  |  | 9.960 |  |  |
| 87 |  |  |  | 12.560 |  |  |
| 88 |  |  |  | 12.110 |  |  |

Table e5: Minute ventilation [l]; Data are median, its lower and its upper limit of 95% confidence interval (CI) of daily mean values of non-survivors and survivors at each day of stay

**Table e6: Tidal volume [ml] per kg of ideal bodyweight**

|  | Non-Survivor | | | Survivor | | |
| --- | --- | --- | --- | --- | --- | --- |
| Day of stay | Median | Lower limit of 95% CI of the median | Upper limit of 95% CI of the median | Median | Lower limit of 95% CI of the median | Upper limit of 95% CI of the median |
| 1 | 5.54 | 5.21 | 5.76 | 5.73 | 5.49 | 5.97 |
| 2 | 5.55 | 5.16 | 5.79 | 5.80 | 5.72 | 5.99 |
| 3 | 5.66 | 5.44 | 5.81 | 5.84 | 5.63 | 6.14 |
| 4 | 5.88 | 5.60 | 6.13 | 6.00 | 5.79 | 6.22 |
| 5 | 6.00 | 5.77 | 6.19 | 6.21 | 5.94 | 6.53 |
| 6 | 5.83 | 5.57 | 6.13 | 6.16 | 5.87 | 6.48 |
| 7 | 5.97 | 5.73 | 6.12 | 6.09 | 5.91 | 6.59 |
| 8 | 5.81 | 5.60 | 6.25 | 6.28 | 5.99 | 6.87 |
| 9 | 5.75 | 5.50 | 6.11 | 6.43 | 6.14 | 7.02 |
| 10 | 6.00 | 5.40 | 6.55 | 6.87 | 6.41 | 7.02 |
| 11 | 5.78 | 5.34 | 6.30 | 6.48 | 6.22 | 7.00 |
| 12 | 5.70 | 5.17 | 6.22 | 6.44 | 6.29 | 6.94 |
| 13 | 5.80 | 5.18 | 6.19 | 6.71 | 6.00 | 7.22 |
| 14 | 5.74 | 5.08 | 6.03 | 6.54 | 5.80 | 7.28 |
| 15 | 5.78 | 5.12 | 6.27 | 6.53 | 5.96 | 7.14 |
| 16 | 5.62 | 4.91 | 6.10 | 6.15 | 5.88 | 7.17 |
| 17 | 5.46 | 5.15 | 6.02 | 6.18 | 5.86 | 6.84 |
| 18 | 5.31 | 4.51 | 5.90 | 6.43 | 6.03 | 6.88 |
| 19 | 5.17 | 4.52 | 5.87 | 6.16 | 5.57 | 6.86 |
| 20 | 5.02 | 3.84 | 6.08 | 5.88 | 5.38 | 6.71 |
| 21 | 4.82 | 2.71 | 5.44 | 5.47 | 5.08 | 6.38 |
| 22 | 5.12 | 2.96 | 5.72 | 5.96 | 5.38 | 6.38 |
| 23 | 4.81 | 1.78 | 5.75 | 5.71 | 5.57 | 6.57 |
| 24 | 4.97 | 1.56 | 5.95 | 5.80 | 5.69 | 6.10 |
| 25 | 4.76 | 1.31 | 6.15 | 5.66 | 5.38 | 5.93 |
| 26 | 4.89 | 1.07 | 6.13 | 5.46 | 5.29 | 6.16 |
| 27 | 4.48 | 0.93 | 6.11 | 5.63 | 5.13 | 6.23 |
| 28 | 5.05 | 1.20 | 6.61 | 5.48 | 4.98 | 6.30 |
| 29 | 4.87 | 1.20 | 6.82 | 5.86 | 4.83 | 7.15 |
| 30 | 4.56 | 1.07 | 6.31 | 6.10 | 5.20 | 6.74 |
| 31 | 5.00 | 1.20 | 5.60 | 5.86 | 5.34 | 7.33 |
| 32 | 5.01 | 1.07 | 6.80 | 6.24 | 5.43 | 6.94 |
| 33 | 5.21 | 1.15 | 7.47 | 6.59 | 5.87 | 7.06 |
| 34 | 5.42 | 1.72 | 6.88 | 6.36 | 5.91 | 7.18 |
| 35 | 5.68 | 4.97 | 7.60 | 5.60 | 4.62 | 7.01 |
| 36 | 5.70 | 4.99 | 7.16 | 5.88 | 4.86 | 6.71 |
| 37 | 5.47 | 4.97 | 6.40 | 5.71 | 4.60 | 6.29 |
| 38 | 4.58 | 4.41 | 6.00 | 5.94 | 5.43 | 6.65 |
| 39 | 5.05 | 4.60 | 6.27 | 6.48 | 5.30 | 7.10 |
| 40 | 4.61 | 3.97 | 6.67 | 6.20 | 5.75 | 7.56 |
| 41 | 4.52 | 3.73 | 6.93 | 7.63 | 5.39 | 8.67 |
| 42 | 5.82 | 4.38 | 8.00 | 6.94 | 5.99 | 8.35 |
| 43 | 6.11 | 4.03 | 7.60 | 6.11 | 4.42 | 10.91 |
| 44 | 6.44 | 4.07 | 7.60 | 6.54 | 5.93 | 9.42 |
| 45 | 5.90 | 4.00 | 7.47 | 7.52 | 6.11 | 9.29 |
| 46 | 5.73 | 3.36 | 7.33 | 7.02 | 5.72 | 8.16 |
| 47 | 4.49 | 3.26 | 5.71 | 6.64 | 5.30 | 8.00 |
| 48 | 4.78 | 3.64 | 5.93 | 5.70 | 5.18 | 10.19 |
| 49 | 4.33 | 3.32 | 5.33 | 6.63 | 5.91 | 11.08 |
| 50 | 4.26 | 2.83 | 5.68 | 6.59 | 5.96 | 9.61 |
| 51 | 5.38 |  |  | 5.96 | 5.65 | 8.27 |
| 52 | 5.45 |  |  | 6.24 | 5.84 | 8.40 |
| 53 | 5.68 |  |  | 5.76 | 5.74 | 8.71 |
| 54 | 5.64 |  |  | 5.60 | 4.82 | 5.85 |
| 55 | 5.28 |  |  | 5.29 | 5.29 | 5.29 |
| 56 | 5.21 |  |  | 5.19 | 5.06 | 5.31 |
| 57 | 4.66 |  |  | 5.68 | 5.41 | 5.95 |
| 58 | 4.91 |  |  | 5.39 | 4.94 | 5.84 |
| 59 | 3.96 |  |  | 5.23 | 5.06 | 5.41 |
| 60 | 3.97 |  |  | 5.32 | 5.18 | 5.46 |
| 61 |  |  |  | 4.88 | 4.59 | 5.18 |
| 62 |  |  |  | 5.09 | 4.77 | 5.41 |
| 63 |  |  |  | 5.14 | 5.00 | 5.29 |
| 64 |  |  |  | 4.90 | 4.51 | 5.29 |
| 65 |  |  |  | 5.28 | 5.26 | 5.29 |
| 66 |  |  |  | 5.15 | 4.82 | 5.47 |
| 67 |  |  |  | 5.48 | 5.19 | 5.76 |
| 68 |  |  |  | 4.80 | 4.55 | 5.06 |
| 69 |  |  |  | 5.87 | 5.03 | 6.71 |
| 70 |  |  |  | 5.23 | 5.05 | 5.41 |
| 71 |  |  |  | 5.04 | 4.47 | 5.60 |
| 72 |  |  |  | 5.38 | 5.12 | 5.65 |
| 73 |  |  |  | 5.46 | 5.15 | 5.76 |
| 74 |  |  |  | 5.29 | 4.92 | 5.65 |
| 75 |  |  |  | 5.37 | 5.21 | 5.53 |
| 76 |  |  |  | 5.56 | 5.47 | 5.65 |
| 77 |  |  |  | 5.16 | 5.06 | 5.26 |
| 78 |  |  |  | 5.27 | 5.25 | 5.29 |
| 79 |  |  |  | 5.74 | 5.24 | 6.24 |
| 80 |  |  |  | 6.30 | 6.01 | 6.59 |
| 81 |  |  |  | 6.10 | 6.00 | 6.20 |
| 82 |  |  |  | 6.45 | 6.31 | 6.59 |
| 83 |  |  |  | 6.32 | 6.12 | 6.51 |
| 84 |  |  |  | 6.09 |  |  |
| 85 |  |  |  | 4.77 |  |  |
| 86 |  |  |  | 4.65 |  |  |
| 87 |  |  |  | 5.37 |  |  |
| 88 |  |  |  | 4.64 |  |  |

Table e6: Tidal volume [ml] per kg of ideal bodyweight; Data are median, its lower and its upper limit of 95% confidence interval (CI) of daily mean values of non-survivors and survivors at each day of stay

**Table e7: Driving pressure**

|  | Non-Survivor | | | Survivor | | |
| --- | --- | --- | --- | --- | --- | --- |
| Day of stay | Median | Lower limit of 95% CI of the median | Upper limit of 95% CI of the median | Median | Lower limit of 95% CI of the median | Upper limit of 95% CI of the median |
| 1 | 14.16 | 13.48 | 15.00 | 13.86 | 12.92 | 14.44 |
| 2 | 14.00 | 13.25 | 14.56 | 12.70 | 12.17 | 13.63 |
| 3 | 14.04 | 13.11 | 14.57 | 12.65 | 12.12 | 13.12 |
| 4 | 14.09 | 13.32 | 14.92 | 12.10 | 11.51 | 12.54 |
| 5 | 14.14 | 13.42 | 15.00 | 11.68 | 11.17 | 12.41 |
| 6 | 15.03 | 13.96 | 15.47 | 11.62 | 11.12 | 12.50 |
| 7 | 14.37 | 13.96 | 15.41 | 11.46 | 10.25 | 12.57 |
| 8 | 14.84 | 14.07 | 15.50 | 11.14 | 9.92 | 12.92 |
| 9 | 15.08 | 14.10 | 15.67 | 11.71 | 10.63 | 12.85 |
| 10 | 15.79 | 14.81 | 16.20 | 12.10 | 10.83 | 13.00 |
| 11 | 15.52 | 14.58 | 16.44 | 11.75 | 10.63 | 12.72 |
| 12 | 15.77 | 15.17 | 16.55 | 12.24 | 11.12 | 13.55 |
| 13 | 16.11 | 15.30 | 16.79 | 13.12 | 12.32 | 15.03 |
| 14 | 16.44 | 15.80 | 17.71 | 12.88 | 11.35 | 14.58 |
| 15 | 16.69 | 15.54 | 17.42 | 13.07 | 11.29 | 13.76 |
| 16 | 16.88 | 15.80 | 17.66 | 12.83 | 11.96 | 14.26 |
| 17 | 16.25 | 15.24 | 17.96 | 12.87 | 11.76 | 14.36 |
| 18 | 16.39 | 15.17 | 17.87 | 12.72 | 11.48 | 14.42 |
| 19 | 16.21 | 15.69 | 18.54 | 14.17 | 12.65 | 15.46 |
| 20 | 16.52 | 15.30 | 17.79 | 14.17 | 12.48 | 17.50 |
| 21 | 16.52 | 15.37 | 17.63 | 15.51 | 12.71 | 16.84 |
| 22 | 16.84 | 15.68 | 19.61 | 14.25 | 13.13 | 15.93 |
| 23 | 17.29 | 15.92 | 19.05 | 14.73 | 13.00 | 16.50 |
| 24 | 16.72 | 15.68 | 18.46 | 14.85 | 14.04 | 17.08 |
| 25 | 16.46 | 15.21 | 18.83 | 15.08 | 14.20 | 17.46 |
| 26 | 17.11 | 15.25 | 19.88 | 15.35 | 13.48 | 18.08 |
| 27 | 16.50 | 13.77 | 17.88 | 15.25 | 12.50 | 16.11 |
| 28 | 16.00 | 14.36 | 20.25 | 14.19 | 13.50 | 16.09 |
| 29 | 17.04 | 11.30 | 20.84 | 15.22 | 12.19 | 17.03 |
| 30 | 17.68 | 13.58 | 21.33 | 15.71 | 13.61 | 17.97 |
| 31 | 17.27 | 14.08 | 24.38 | 16.00 | 13.33 | 19.11 |
| 32 | 17.52 | 15.17 | 27.04 | 15.73 | 12.80 | 18.08 |
| 33 | 18.12 | 15.79 | 20.66 | 13.00 | 11.72 | 16.31 |
| 34 | 16.83 | 16.18 | 17.82 | 15.21 | 11.67 | 17.30 |
| 35 | 17.04 | 17.01 | 19.83 | 16.54 | 12.47 | 18.04 |
| 36 | 17.88 | 17.34 | 18.68 | 17.00 | 10.89 | 18.57 |
| 37 | 17.54 | 16.79 | 20.12 | 17.40 | 11.00 | 19.48 |
| 38 | 17.58 | 17.21 | 20.90 | 18.53 | 11.83 | 21.17 |
| 39 | 18.06 | 15.23 | 20.74 | 17.33 | 8.80 | 19.88 |
| 40 | 17.64 | 12.62 | 22.36 | 16.96 | 13.68 | 19.79 |
| 41 | 17.41 | 14.96 | 23.56 | 16.59 | 13.85 | 20.70 |
| 42 | 17.04 | 14.50 | 22.54 | 18.89 | 12.13 | 21.61 |
| 43 | 13.76 | 12.91 | 22.42 | 19.56 | 13.14 | 23.17 |
| 44 | 14.69 | 13.67 | 23.40 | 18.69 | 13.22 | 23.67 |
| 45 | 14.74 | 12.96 | 20.96 | 18.19 | 15.79 | 22.50 |
| 46 | 15.15 | 12.46 | 21.56 | 19.25 | 17.50 | 20.46 |
| 47 | 19.28 | 16.22 | 22.33 | 17.32 | 14.88 | 21.22 |
| 48 | 18.58 | 15.15 | 22.00 | 17.90 | 17.82 | 26.77 |
| 49 | 18.44 | 14.67 | 22.20 | 19.72 | 17.04 | 26.12 |
| 50 | 20.43 | 13.48 | 27.38 | 17.12 | 15.46 | 27.50 |
| 51 | 12.31 |  |  | 20.26 | 16.00 | 25.88 |
| 52 | 13.65 |  |  | 19.46 | 16.13 | 26.68 |
| 53 | 12.64 |  |  | 16.63 | 10.85 | 18.86 |
| 54 | 13.79 |  |  | 21.29 | 15.72 | 21.30 |
| 55 | 16.27 |  |  | 17.58 | 12.78 | 22.38 |
| 56 | 16.30 |  |  | 16.46 | 10.55 | 22.36 |
| 57 | 17.01 |  |  | 18.83 | 14.70 | 22.96 |
| 58 | 17.93 |  |  | 18.14 | 13.95 | 22.32 |
| 59 | 17.93 |  |  | 16.52 | 10.78 | 22.25 |
| 60 | 19.00 |  |  | 14.92 | 9.88 | 19.96 |
| 61 |  |  |  | 14.45 | 8.82 | 20.08 |
| 62 |  |  |  | 14.79 | 9.58 | 20.00 |
| 63 |  |  |  | 14.18 | 9.32 | 19.04 |
| 64 |  |  |  | 14.33 | 9.78 | 18.88 |
| 65 |  |  |  | 15.68 | 12.28 | 19.08 |
| 66 |  |  |  | 14.45 | 9.47 | 19.42 |
| 67 |  |  |  | 19.00 | 18.00 | 20.00 |
| 68 |  |  |  | 17.70 | 17.14 | 18.25 |
| 69 |  |  |  | 14.34 | 13.21 | 15.46 |
| 70 |  |  |  | 16.75 | 14.50 | 19.00 |
| 71 |  |  |  | 17.05 | 16.26 | 17.83 |
| 72 |  |  |  | 18.74 | 17.96 | 19.52 |
| 73 |  |  |  | 16.92 | 15.75 | 18.08 |
| 74 |  |  |  | 16.48 | 15.64 | 17.32 |
| 75 |  |  |  | 16.06 | 15.07 | 17.04 |
| 76 |  |  |  | 16.04 | 15.15 | 16.92 |
| 77 |  |  |  | 15.01 | 13.52 | 16.50 |
| 78 |  |  |  | 15.96 | 15.83 | 16.08 |
| 79 |  |  |  | 17.44 | 17.08 | 17.80 |
| 80 |  |  |  | 14.87 | 12.15 | 17.59 |
| 81 |  |  |  | 15.41 | 14.38 | 16.44 |
| 82 |  |  |  | 16.11 | 13.88 | 18.33 |
| 83 |  |  |  | 17.07 | 14.57 | 19.56 |
| 84 |  |  |  | 18.00 |  |  |
| 85 |  |  |  | 15.71 |  |  |
| 86 |  |  |  | 19.50 |  |  |
| 87 |  |  |  | 17.79 |  |  |
| 88 |  |  |  | 9.33 |  |  |

Table e7: Driving pressure [mbar]; Data are median, its lower and its upper limit of 95% confidence interval (CI) of daily mean values of non-survivors and survivors at each day of stay

**Table e8: C-reactive protein**

|  | Non-Survivor | | | Survivor | | |
| --- | --- | --- | --- | --- | --- | --- |
| Day of stay | Median | Lower limit of 95% CI of the median | Upper limit of 95% CI of the median | Median | Lower limit of 95% CI of the median | Upper limit of 95% CI of the median |
| 1 | 182,60 | 204,10 | 159,55 | 153,65 | 173,90 | 131,30 |
| 2 | 183,50 | 192,05 | 165,20 | 140,45 | 186,50 | 120,95 |
| 3 | 147,70 | 176,65 | 126,10 | 117,40 | 152,40 | 95,80 |
| 4 | 137,85 | 156,40 | 109,10 | 94,30 | 130,90 | 83,00 |
| 5 | 143,60 | 162,20 | 120,00 | 89,00 | 102,30 | 69,60 |
| 6 | 135,80 | 155,80 | 119,20 | 78,10 | 92,40 | 66,00 |
| 7 | 141,90 | 156,10 | 120,60 | 76,35 | 95,20 | 62,70 |
| 8 | 155,93 | 178,20 | 121,80 | 75,63 | 95,70 | 61,00 |
| 9 | 143,45 | 169,30 | 121,45 | 71,40 | 95,50 | 55,60 |
| 10 | 142,70 | 165,20 | 116,30 | 89,90 | 102,30 | 73,90 |
| 11 | 145,75 | 181,40 | 122,50 | 80,80 | 98,10 | 68,10 |
| 12 | 172,70 | 183,00 | 151,30 | 82,20 | 98,90 | 63,80 |
| 13 | 173,00 | 214,20 | 145,00 | 84,05 | 109,60 | 50,90 |
| 14 | 166,50 | 203,30 | 129,20 | 87,95 | 116,00 | 52,50 |
| 15 | 153,95 | 214,10 | 121,40 | 87,10 | 102,40 | 53,80 |
| 16 | 159,95 | 201,40 | 115,90 | 89,20 | 109,50 | 61,00 |
| 17 | 153,10 | 174,60 | 121,80 | 77,00 | 98,00 | 59,20 |
| 18 | 140,90 | 192,20 | 101,40 | 74,15 | 99,50 | 54,60 |
| 19 | 120,40 | 185,80 | 78,00 | 65,80 | 92,30 | 51,20 |
| 20 | 114,15 | 203,20 | 86,60 | 73,90 | 100,60 | 50,70 |
| 21 | 135,50 | 194,60 | 99,20 | 72,90 | 117,00 | 38,70 |
| 22 | 151,69 | 207,80 | 114,90 | 57,40 | 115,60 | 37,00 |
| 23 | 127,15 | 208,50 | 88,80 | 64,10 | 100,40 | 38,90 |
| 24 | 113,50 | 188,70 | 68,80 | 75,20 | 97,80 | 37,20 |
| 25 | 107,15 | 224,70 | 62,60 | 62,40 | 92,00 | 30,50 |
| 26 | 133,80 | 267,20 | 48,40 | 59,90 | 107,30 | 33,90 |
| 27 | 83,75 | 265,10 | 64,80 | 77,00 | 105,40 | 40,70 |
| 28 | 102,55 | 234,60 | 34,90 | 65,20 | 89,80 | 52,30 |
| 29 | 52,50 | 167,50 | 28,50 | 63,40 | 86,40 | 54,30 |
| 30 | 59,40 | 154,90 | 20,00 | 67,30 | 102,50 | 50,30 |
| 31 | 78,40 | 155,90 | 33,30 | 67,50 | 154,90 | 43,20 |
| 32 | 91,50 | 145,90 | 51,90 | 61,60 | 192,30 | 34,60 |
| 33 | 101,40 | 328,70 | 82,40 | 78,50 | 109,60 | 48,00 |
| 34 | 114,60 | 212,90 | 89,20 | 84,15 | 102,95 | 67,00 |
| 35 | 113,55 | 124,30 | 71,80 | 73,70 | 126,60 | 40,50 |
| 36 | 114,70 | 230,40 | 54,10 | 61,40 | 137,90 | 52,90 |
| 37 | 115,70 | 245,20 | 55,20 | 56,90 | 118,00 | 51,90 |
| 38 | 87,20 | 269,70 | 56,50 | 64,00 | 107,90 | 41,80 |
| 39 | 69,65 | 314,60 | 24,00 | 56,95 | 97,10 | 25,90 |
| 40 | 68,25 | 311,40 | 21,40 | 57,65 | 151,40 | 25,60 |
| 41 | 90,50 | 237,10 | 52,40 | 71,65 | 137,90 | 29,40 |
| 42 | 78,70 | 90,90 | 66,50 | 59,15 | 161,20 | 30,70 |
| 43 | 73,30 | 81,90 | 64,70 | 62,30 | 164,40 | 19,60 |
| 44 | 60,15 | 70,20 | 50,10 | 61,70 | 130,40 | 21,00 |
| 45 | 62,85 | 80,80 | 44,90 | 54,80 | 100,40 | 10,40 |
| 46 | 65,30 | 75,20 | 55,40 | 48,55 | 137,30 | 7,90 |
| 47 | 60,30 | 62,90 | 57,70 | 60,35 | 284,55 | 37,40 |
| 48 | 77,90 | 78,10 | 77,70 | 101,10 | 225,40 | 39,40 |
| 49 | 119,65 | 131,80 | 107,50 | 113,20 | 151,40 | 74,40 |
| 50 | 178,80 |  |  | 87,70 | 133,70 | 67,60 |
| 51 | 226,00 |  |  | 64,20 | 114,10 | 56,00 |
| 52 | 218,30 |  |  | 93,90 | 123,20 | 49,10 |
| 53 | 258,10 |  |  | 118,00 | 279,10 | 32,40 |
| 54 | 264,90 |  |  | 125,30 | 213,20 | 108,60 |
| 55 | 333,60 |  |  | 147,80 | 158,80 | 89,30 |
| 56 | 332,70 |  |  | 116,35 | 137,40 | 95,30 |
| 57 | 323,50 |  |  | 71,20 | 94,70 | 47,70 |
| 58 | 338,20 |  |  | 65,70 | 105,80 | 25,60 |
| 59 | 222,70 |  |  | 78,15 | 142,30 | 14,00 |
| 60 | 180,40 |  |  | 73,50 | 136,00 | 11,00 |
| 61 |  |  |  | 60,45 | 112,90 | 8,00 |
| 62 |  |  |  | 74,50 | 143,50 | 5,50 |
| 63 |  |  |  | 81,45 | 157,50 | 5,40 |
| 64 |  |  |  | 71,35 | 130,20 | 12,50 |
| 65 |  |  |  | 68,05 | 112,20 | 23,90 |
| 66 |  |  |  | 170,50 | 204,30 | 136,70 |
| 67 |  |  |  | 313,95 | 328,20 | 299,70 |
| 68 |  |  |  | 289,65 | 385,60 | 193,70 |
| 69 |  |  |  | 262,05 | 427,00 | 97,10 |
| 70 |  |  |  | 195,25 | 319,50 | 71,00 |
| 71 |  |  |  | 98,05 | 150,20 | 45,90 |
| 72 |  |  |  | 99,95 | 164,80 | 35,10 |
| 73 |  |  |  | 190,15 | 350,40 | 29,90 |
| 74 |  |  |  | 166,85 | 307,60 | 26,10 |
| 75 |  |  |  | 104,30 | 175,20 | 33,40 |
| 76 |  |  |  | 113,90 |  |  |
| 77 |  |  |  | 66,75 | 107,40 | 26,10 |
| 78 |  |  |  | 122,80 |  |  |
| 79 |  |  |  | 61,30 | 103,20 | 19,40 |
| 80 |  |  |  | 63,20 | 97,40 | 29,00 |
| 81 |  |  |  | 68,75 | 108,70 | 28,80 |
| 82 |  |  |  | 76,55 | 106,00 | 47,10 |
| 83 |  |  |  | 67,80 | 89,60 | 46,00 |
| 84 |  |  |  | 60,00 |  |  |
| 85 |  |  |  | 42,40 |  |  |
| 86 |  |  |  | 35,00 |  |  |
| 87 |  |  |  | 29,90 |  |  |
| 88 |  |  |  | 61,90 |  |  |
| 89 |  |  |  | 65,30 |  |  |

Table e8: C-reactive protein [mg/l]; Data are median, its lower and its upper limit of 95% confidence interval (CI) of daily mean values of non-survivors and survivors at each day of stay

**Table e9: Leukocytes**

|  | Non-Survivor | | | Survivor | | |
| --- | --- | --- | --- | --- | --- | --- |
| Day of stay | Median | Lower limit of 95% CI of the median | Upper limit of 95% CI of the median | Median | Lower limit of 95% CI of the median | Upper limit of 95% CI of the median |
| 1 | 13,05 | 14,95 | 11,88 | 12,99 | 14,12 | 10,64 |
| 2 | 13,06 | 14,08 | 11,79 | 12,80 | 13,65 | 10,96 |
| 3 | 12,82 | 14,43 | 11,77 | 11,81 | 13,11 | 10,69 |
| 4 | 13,45 | 14,82 | 12,09 | 11,65 | 12,96 | 10,98 |
| 5 | 13,48 | 14,56 | 12,22 | 12,73 | 13,79 | 11,20 |
| 6 | 14,14 | 15,30 | 12,94 | 13,18 | 15,21 | 11,73 |
| 7 | 13,15 | 14,77 | 12,52 | 12,72 | 14,94 | 11,71 |
| 8 | 13,84 | 14,82 | 12,29 | 12,73 | 14,15 | 11,80 |
| 9 | 13,99 | 16,49 | 11,23 | 11,45 | 12,54 | 10,33 |
| 10 | 13,79 | 15,81 | 11,64 | 10,83 | 12,74 | 9,37 |
| 11 | 12,92 | 16,09 | 11,26 | 10,63 | 12,22 | 9,18 |
| 12 | 11,90 | 13,37 | 10,22 | 10,36 | 12,22 | 8,95 |
| 13 | 11,02 | 12,55 | 9,42 | 9,39 | 10,77 | 8,57 |
| 14 | 9,91 | 11,82 | 9,22 | 10,19 | 11,25 | 8,46 |
| 15 | 10,84 | 11,78 | 9,72 | 10,28 | 11,17 | 9,01 |
| 16 | 10,85 | 11,59 | 9,89 | 9,94 | 11,52 | 8,62 |
| 17 | 10,83 | 11,50 | 9,09 | 10,08 | 12,62 | 9,19 |
| 18 | 9,67 | 12,38 | 8,69 | 10,68 | 12,74 | 9,53 |
| 19 | 10,48 | 14,57 | 8,47 | 11,23 | 12,89 | 9,79 |
| 20 | 11,84 | 15,51 | 9,01 | 11,71 | 13,34 | 10,01 |
| 21 | 10,47 | 14,74 | 8,96 | 11,87 | 13,11 | 9,83 |
| 22 | 10,39 | 14,06 | 9,74 | 11,25 | 14,32 | 10,33 |
| 23 | 10,65 | 13,70 | 7,38 | 11,53 | 14,25 | 10,48 |
| 24 | 9,17 | 14,12 | 6,60 | 11,98 | 14,13 | 10,42 |
| 25 | 8,67 | 12,53 | 5,09 | 12,16 | 13,94 | 10,70 |
| 26 | 11,71 | 14,32 | 6,32 | 12,79 | 18,61 | 10,78 |
| 27 | 11,49 | 15,99 | 8,35 | 13,71 | 16,15 | 11,28 |
| 28 | 9,53 | 13,56 | 7,22 | 12,97 | 14,67 | 11,05 |
| 29 | 8,14 | 17,09 | 6,32 | 12,69 | 15,99 | 11,25 |
| 30 | 9,03 | 15,20 | 6,98 | 13,40 | 18,00 | 11,77 |
| 31 | 8,71 | 12,91 | 6,32 | 14,77 | 19,21 | 11,14 |
| 32 | 8,93 | 13,78 | 5,79 | 13,83 | 21,47 | 11,10 |
| 33 | 8,16 | 22,88 | 6,83 | 14,60 | 20,04 | 12,39 |
| 34 | 9,19 | 16,16 | 6,54 | 16,91 | 18,19 | 11,42 |
| 35 | 12,32 | 15,25 | 9,28 | 15,42 | 21,61 | 13,47 |
| 36 | 12,93 | 15,05 | 8,03 | 15,15 | 19,29 | 13,92 |
| 37 | 11,51 | 15,45 | 9,40 | 15,03 | 18,81 | 13,31 |
| 38 | 15,47 | 17,21 | 9,51 | 14,50 | 19,31 | 11,81 |
| 39 | 14,38 | 16,63 | 9,74 | 13,95 | 16,24 | 11,20 |
| 40 | 16,08 | 18,65 | 10,10 | 14,01 | 19,42 | 11,42 |
| 41 | 14,03 | 14,64 | 10,08 | 14,80 | 19,30 | 9,33 |
| 42 | 11,80 | 16,02 | 7,58 | 15,59 | 18,77 | 9,16 |
| 43 | 12,13 | 16,79 | 7,46 | 11,94 | 16,14 | 8,13 |
| 44 | 11,38 | 16,09 | 6,67 | 10,42 | 13,49 | 8,98 |
| 45 | 10,79 | 14,84 | 6,74 | 11,22 | 14,64 | 8,10 |
| 46 | 10,61 | 12,47 | 8,74 | 10,03 | 15,38 | 6,44 |
| 47 | 9,03 | 9,56 | 8,50 | 9,80 | 13,11 | 7,35 |
| 48 | 10,57 | 13,85 | 7,29 | 10,99 | 11,95 | 8,18 |
| 49 | 12,06 | 13,57 | 10,54 | 10,82 | 16,29 | 8,46 |
| 50 | 10,49 |  |  | 9,24 | 13,52 | 8,03 |
| 51 | 10,81 |  |  | 8,71 | 16,87 | 8,01 |
| 52 | 10,01 |  |  | 11,00 | 22,18 | 8,22 |
| 53 | 9,24 |  |  | 10,32 | 22,08 | 7,70 |
| 54 | 9,50 |  |  | 8,40 | 20,96 | 6,13 |
| 55 | 12,26 |  |  | 9,10 | 18,67 | 5,48 |
| 56 | 10,81 |  |  | 6,13 | 7,02 | 5,24 |
| 57 | 12,21 |  |  | 8,61 | 8,92 | 8,30 |
| 58 | 9,27 |  |  | 9,38 | 10,60 | 8,15 |
| 59 | 11,82 |  |  | 9,55 | 10,84 | 8,25 |
| 60 | 11,39 |  |  | 10,32 | 11,63 | 9,01 |
| 61 |  |  |  | 10,82 | 12,39 | 9,25 |
| 62 |  |  |  | 9,95 | 12,18 | 7,71 |
| 63 |  |  |  | 10,79 | 10,95 | 10,62 |
| 64 |  |  |  | 10,49 | 10,67 | 10,30 |
| 65 |  |  |  | 8,13 | 8,20 | 8,05 |
| 66 |  |  |  | 10,17 | 10,67 | 9,66 |
| 67 |  |  |  | 9,39 | 11,32 | 7,46 |
| 68 |  |  |  | 10,96 | 16,63 | 5,28 |
| 69 |  |  |  | 11,99 | 18,28 | 5,70 |
| 70 |  |  |  | 10,06 | 13,81 | 6,30 |
| 71 |  |  |  | 11,12 | 16,00 | 6,23 |
| 72 |  |  |  | 11,25 | 15,35 | 7,15 |
| 73 |  |  |  | 17,14 | 27,12 | 7,15 |
| 74 |  |  |  | 15,19 | 23,20 | 7,18 |
| 75 |  |  |  | 13,37 | 19,25 | 7,49 |
| 76 |  |  |  | 20,33 |  |  |
| 77 |  |  |  | 13,61 | 20,67 | 6,55 |
| 78 |  |  |  | 18,12 |  |  |
| 79 |  |  |  | 10,55 | 13,90 | 7,20 |
| 80 |  |  |  | 10,21 | 13,27 | 7,14 |
| 81 |  |  |  | 10,25 | 10,30 | 10,20 |
| 82 |  |  |  | 8,45 | 8,95 | 7,95 |
| 83 |  |  |  | 7,37 | 8,69 | 6,05 |
| 84 |  |  |  | 10,50 |  |  |
| 85 |  |  |  | 9,99 |  |  |
| 86 |  |  |  | 9,77 |  |  |
| 87 |  |  |  | 10,16 |  |  |
| 88 |  |  |  | 8,20 |  |  |
| 89 |  |  |  | 6,24 |  |  |

Table e9: Leucocytes [GPt/L]; Data are median, its lower and its upper limit of 95% confidence interval (CI) of daily mean values of non-survivors and survivors at each day of stay

**Table e10: PaO2/FiO2 ratio**

|  | Non-Survivor | | | Survivor | | |
| --- | --- | --- | --- | --- | --- | --- |
| Day of stay | Median | Lower limit of 95% CI of the median | Upper limit of 95% CI of the median | Median | Lower limit of 95% CI of the median | Upper limit of 95% CI of the median |
| 1 | 135,00 | 142,50 | 124,29 | 144,78 | 149,32 | 141,56 |
| 2 | 143,81 | 151,88 | 137,73 | 165,00 | 181,61 | 155,00 |
| 3 | 140,63 | 150,00 | 130,71 | 172,19 | 185,00 | 164,38 |
| 4 | 140,00 | 152,50 | 135,00 | 180,47 | 196,07 | 171,35 |
| 5 | 135,00 | 147,75 | 130,63 | 175,45 | 183,75 | 163,93 |
| 6 | 140,18 | 154,09 | 130,23 | 182,50 | 192,50 | 168,75 |
| 7 | 135,27 | 151,25 | 126,92 | 193,75 | 205,00 | 184,38 |
| 8 | 141,75 | 157,50 | 132,95 | 189,32 | 202,50 | 174,64 |
| 9 | 135,94 | 151,07 | 128,18 | 196,25 | 211,67 | 180,75 |
| 10 | 133,48 | 144,38 | 125,36 | 192,19 | 205,50 | 181,25 |
| 11 | 135,38 | 147,00 | 129,38 | 200,36 | 213,75 | 181,73 |
| 12 | 128,75 | 153,75 | 118,75 | 199,69 | 216,67 | 185,00 |
| 13 | 137,44 | 146,25 | 118,13 | 211,88 | 217,50 | 190,83 |
| 14 | 125,72 | 143,44 | 113,57 | 203,57 | 226,88 | 174,55 |
| 15 | 127,89 | 143,33 | 118,13 | 203,75 | 228,75 | 184,29 |
| 16 | 133,13 | 155,00 | 126,14 | 215,19 | 233,57 | 197,50 |
| 17 | 135,00 | 153,21 | 124,29 | 212,92 | 225,00 | 181,67 |
| 18 | 122,14 | 139,17 | 109,38 | 201,09 | 234,64 | 171,92 |
| 19 | 123,63 | 145,63 | 113,25 | 202,50 | 236,25 | 176,25 |
| 20 | 116,25 | 151,67 | 104,06 | 207,27 | 242,14 | 178,75 |
| 21 | 128,25 | 140,63 | 103,64 | 211,25 | 245,36 | 183,75 |
| 22 | 121,35 | 133,33 | 110,00 | 226,72 | 251,79 | 188,86 |
| 23 | 123,41 | 138,75 | 105,00 | 216,43 | 246,25 | 180,00 |
| 24 | 130,34 | 151,07 | 104,06 | 229,29 | 247,50 | 176,25 |
| 25 | 120,13 | 139,17 | 108,00 | 241,25 | 266,25 | 185,63 |
| 26 | 113,66 | 159,55 | 105,58 | 234,75 | 252,00 | 209,38 |
| 27 | 135,68 | 175,83 | 123,75 | 233,75 | 270,00 | 195,00 |
| 28 | 129,20 | 186,00 | 106,50 | 236,25 | 270,00 | 183,75 |
| 29 | 123,75 | 177,00 | 114,38 | 227,65 | 267,50 | 170,63 |
| 30 | 130,71 | 186,67 | 119,17 | 217,32 | 253,13 | 179,17 |
| 31 | 131,41 | 186,25 | 98,44 | 201,25 | 249,38 | 180,83 |
| 32 | 120,78 | 219,38 | 83,44 | 211,13 | 240,00 | 168,75 |
| 33 | 143,75 | 209,06 | 105,63 | 203,25 | 255,00 | 168,75 |
| 34 | 148,93 | 201,14 | 111,92 | 194,63 | 233,75 | 178,93 |
| 35 | 136,88 | 199,09 | 75,00 | 191,25 | 246,25 | 177,95 |
| 36 | 140,36 | 213,13 | 140,36 | 182,59 | 260,00 | 153,75 |
| 37 | 144,38 | 203,86 | 94,50 | 182,38 | 243,21 | 166,88 |
| 38 | 138,21 | 236,25 | 81,25 | 212,26 | 277,50 | 166,25 |
| 39 | 143,44 | 230,36 | 82,50 | 206,25 | 247,50 | 189,17 |
| 40 | 144,03 | 242,50 | 72,00 | 241,25 | 304,17 | 190,50 |
| 41 | 125,83 | 133,93 | 68,18 | 220,93 | 354,38 | 176,25 |
| 42 | 150,38 | 165,75 | 135,00 | 212,68 | 301,50 | 195,00 |
| 43 | 142,76 | 160,83 | 124,69 | 220,78 | 285,00 | 212,50 |
| 44 | 144,11 | 146,79 | 141,43 | 234,02 | 286,88 | 202,50 |
| 45 | 167,71 | 171,67 | 163,75 | 243,57 | 272,50 | 202,97 |
| 46 | 156,16 | 160,31 | 152,00 | 246,25 | 296,79 | 216,00 |
| 47 | 152,84 | 178,93 | 126,75 | 225,82 | 298,13 | 193,13 |
| 48 | 141,15 | 181,67 | 100,63 | 228,80 | 289,50 | 169,69 |
| 49 | 123,61 | 161,25 | 85,96 | 223,50 | 285,00 | 176,25 |
| 50 | 144,64 |  |  | 279,38 | 326,79 | 270,00 |
| 51 | 150,83 |  |  | 265,71 | 290,36 | 242,14 |
| 52 | 129,38 |  |  | 273,75 | 309,38 | 252,86 |
| 53 | 111,00 |  |  | 285,00 | 340,00 | 237,50 |
| 54 | 111,43 |  |  | 255,36 | 300,00 | 203,75 |
| 55 | 87,95 |  |  | 226,25 | 268,13 | 215,00 |
| 56 | 103,33 |  |  | 201,25 | 216,25 | 186,25 |
| 57 | 70,00 |  |  | 215,94 | 223,13 | 208,75 |
| 58 | 64,17 |  |  | 224,38 | 238,75 | 210,00 |
| 59 | 62,50 |  |  | 243,00 | 253,50 | 232,50 |
| 60 | 47,50 |  |  | 239,82 | 249,64 | 230,00 |
| 61 | 45,00 |  |  | 241,88 | 270,00 | 213,75 |
| 62 |  |  |  | 315,63 | 350,00 | 281,25 |
| 63 |  |  |  | 279,00 | 315,00 | 243,00 |
| 64 |  |  |  | 276,25 | 303,75 | 248,75 |
| 65 |  |  |  | 283,25 | 301,50 | 265,00 |
| 66 |  |  |  | 224,20 | 228,75 | 219,64 |
| 67 |  |  |  | 178,13 | 180,00 | 176,25 |
| 68 |  |  |  | 182,81 |  |  |
| 69 |  |  |  | 174,00 |  |  |
| 70 |  |  |  | 212,50 | 230,00 | 195,00 |
| 71 |  |  |  | 171,43 |  |  |
| 72 |  |  |  | 136,41 | 145,31 | 127,50 |
| 73 |  |  |  | 216,25 |  |  |
| 74 |  |  |  | 247,50 |  |  |
| 75 |  |  |  | 258,75 | 262,50 | 255,00 |
| 76 |  |  |  | 270,54 | 277,50 | 263,57 |
| 77 |  |  |  | 240,00 |  |  |
| 78 |  |  |  | 245,00 |  |  |
| 79 |  |  |  | 194,17 |  |  |
| 80 |  |  |  | 236,25 |  |  |
| 81 |  |  |  | 256,88 |  |  |
| 82 |  |  |  | 249,38 |  |  |
| 83 |  |  |  | 268,13 |  |  |
| 84 |  |  |  | 235,50 |  |  |
| 85 |  |  |  | 234,38 |  |  |
| 86 |  |  |  | 296,79 |  |  |
| 87 |  |  |  | 208,13 |  |  |
| 88 |  |  |  | 305,63 |  |  |
| 89 |  |  |  | 252,50 |  |  |

Table e10: PaO2/FiO2 ratio [mmHg]; Data are median, its lower and its upper limit of 95% confidence interval (CI) of daily mean values of non-survivors and survivors at each day of stay

**Table e11: Procalcitonin**

|  | Non-Survivor | | | Survivor | | |
| --- | --- | --- | --- | --- | --- | --- |
| Day of stay | Median | Lower limit of 95% CI of the median | Upper limit of 95% CI of the median | Median | Lower limit of 95% CI of the median | Upper limit of 95% CI of the median |
| 1 | 1,70 | 2,45 | 1,02 | 0,74 | 1,22 | 0,52 |
| 2 | 1,63 | 2,46 | 1,32 | 0,64 | 0,98 | 0,46 |
| 3 | 1,36 | 2,17 | 1,10 | 0,56 | 0,81 | 0,40 |
| 4 | 1,24 | 1,69 | 0,93 | 0,45 | 0,83 | 0,40 |
| 5 | 1,21 | 2,02 | 0,86 | 0,45 | 0,68 | 0,34 |
| 6 | 1,10 | 1,67 | 0,85 | 0,40 | 0,55 | 0,31 |
| 7 | 1,25 | 1,58 | 0,99 | 0,42 | 0,55 | 0,32 |
| 8 | 1,65 | 1,90 | 1,08 | 0,39 | 0,58 | 0,32 |
| 9 | 1,42 | 1,83 | 1,19 | 0,35 | 0,65 | 0,26 |
| 10 | 1,26 | 1,78 | 1,08 | 0,39 | 0,66 | 0,24 |
| 11 | 1,44 | 1,88 | 1,10 | 0,55 | 0,66 | 0,39 |
| 12 | 1,85 | 2,49 | 1,26 | 0,54 | 0,66 | 0,34 |
| 13 | 2,06 | 2,98 | 1,15 | 0,54 | 0,82 | 0,30 |
| 14 | 1,93 | 2,86 | 1,36 | 0,57 | 0,80 | 0,36 |
| 15 | 2,12 | 3,17 | 1,27 | 0,66 | 1,48 | 0,36 |
| 16 | 2,24 | 3,26 | 1,27 | 0,64 | 1,35 | 0,37 |
| 17 | 1,94 | 3,47 | 1,22 | 0,76 | 1,33 | 0,51 |
| 18 | 2,86 | 4,84 | 1,40 | 0,54 | 1,10 | 0,39 |
| 19 | 4,11 | 5,28 | 2,36 | 0,94 | 1,61 | 0,56 |
| 20 | 3,56 | 5,83 | 1,89 | 1,13 | 1,72 | 0,60 |
| 21 | 3,41 | 5,39 | 2,48 | 1,07 | 1,57 | 0,56 |
| 22 | 3,62 | 6,66 | 2,72 | 1,09 | 2,05 | 0,60 |
| 23 | 4,38 | 7,77 | 1,14 | 0,81 | 1,71 | 0,30 |
| 24 | 3,95 | 7,18 | 1,49 | 0,97 | 2,31 | 0,64 |
| 25 | 3,39 | 7,47 | 0,95 | 0,78 | 2,33 | 0,58 |
| 26 | 2,53 | 10,50 | 0,50 | 0,96 | 2,60 | 0,55 |
| 27 | 3,48 | 8,67 | 0,46 | 0,82 | 2,26 | 0,43 |
| 28 | 2,93 | 8,95 | 0,86 | 1,24 | 1,83 | 0,40 |
| 29 | 1,87 | 7,29 | 0,67 | 1,18 | 2,19 | 0,44 |
| 30 | 2,97 | 5,43 | 0,96 | 1,26 | 1,84 | 0,34 |
| 31 | 2,35 | 4,80 | 0,55 | 1,18 | 2,15 | 0,31 |
| 32 | 2,97 | 5,51 | 0,46 | 1,33 | 2,56 | 0,47 |
| 33 | 2,56 | 2,94 | 0,40 | 1,39 | 2,89 | 0,89 |
| 34 | 2,01 | 3,35 | 0,47 | 1,54 | 2,50 | 0,93 |
| 35 | 2,06 | 7,95 | 0,97 | 1,55 | 2,70 | 0,82 |
| 36 | 4,12 | 7,40 | 1,21 | 1,58 | 2,66 | 0,69 |
| 37 | 2,59 | 6,74 | 1,50 | 1,54 | 2,50 | 0,29 |
| 38 | 3,37 | 8,62 | 0,50 | 1,25 | 1,94 | 0,63 |
| 39 | 2,94 | 9,35 | 0,52 | 1,08 | 1,59 | 0,57 |
| 40 | 2,42 | 7,51 | 0,87 | 0,87 | 1,42 | 0,62 |
| 41 | 1,20 | 6,96 | 0,85 | 0,92 | 2,12 | 0,90 |
| 42 | 0,86 | 1,20 | 0,51 | 1,00 | 1,44 | 0,83 |
| 43 | 0,78 | 1,11 | 0,45 | 0,91 | 1,15 | 0,37 |
| 44 | 0,78 | 1,14 | 0,42 | 0,90 | 1,07 | 0,66 |
| 45 | 0,92 | 1,25 | 0,58 | 0,82 | 0,85 | 0,54 |
| 46 | 1,14 | 1,35 | 0,93 | 0,78 | 0,92 | 0,40 |
| 47 | 1,13 | 1,23 | 1,02 | 1,02 | 1,83 | 0,29 |
| 48 | 0,91 | 1,04 | 0,77 | 2,12 | 32,40 | 0,64 |
| 49 | 1,19 | 1,30 | 1,08 | 1,87 | 16,10 | 1,21 |
| 50 | 1,18 |  |  | 1,89 | 7,78 | 1,14 |
| 51 | 1,71 |  |  | 1,20 | 4,14 | 0,97 |
| 52 | 1,92 |  |  | 1,66 | 1,92 | 0,78 |
| 53 | 1,96 |  |  | 0,66 | 6,89 | 0,53 |
| 54 | 2,66 |  |  | 0,55 | 4,68 | 0,50 |
| 55 | 6,59 |  |  | 1,58 | 2,66 | 0,49 |
| 56 | 11,60 |  |  | 1,04 | 1,66 | 0,42 |
| 57 | 16,30 |  |  | 0,91 |  |  |
| 58 | 26,00 |  |  | 0,60 |  |  |
| 59 | 19,30 |  |  |  |  |  |
| 60 | 13,30 |  |  | 0,38 |  |  |
| 61 |  |  |  | 0,31 | 0,33 | 0,29 |
| 62 |  |  |  | 0,24 |  |  |
| 63 |  |  |  | 0,28 |  |  |
| 64 |  |  |  | 0,40 | 0,47 | 0,32 |
| 65 |  |  |  | 0,42 | 0,45 | 0,39 |
| 66 |  |  |  | 0,77 | 0,82 | 0,71 |
| 67 |  |  |  | 17,44 | 29,36 | 5,52 |
| 68 |  |  |  | 81,10 | 132,40 | 29,80 |
| 69 |  |  |  | 51,90 | 90,00 | 13,79 |
| 70 |  |  |  | 43,26 | 80,30 | 6,21 |
| 71 |  |  |  | 23,85 | 44,80 | 2,90 |
| 72 |  |  |  | 16,35 | 31,00 | 1,70 |
| 73 |  |  |  | 13,43 | 25,80 | 1,06 |
| 74 |  |  |  | 14,26 | 27,60 | 0,92 |
| 75 |  |  |  | 11,41 | 21,70 | 1,11 |
| 76 |  |  |  | 11,90 |  |  |
| 77 |  |  |  | 4,20 | 7,64 | 0,75 |
| 78 |  |  |  | 4,65 |  |  |
| 79 |  |  |  | 0,58 |  |  |
| 80 |  |  |  | 1,16 | 1,74 | 0,58 |
| 81 |  |  |  | 0,83 | 1,10 | 0,56 |
| 82 |  |  |  | 0,95 | 1,16 | 0,74 |
| 83 |  |  |  | 0,99 | 1,43 | 0,55 |
| 84 |  |  |  | 0,35 |  |  |
| 85 |  |  |  | 0,34 |  |  |
| 86 |  |  |  |  |  |  |
| 87 |  |  |  | 0,29 |  |  |
| 88 |  |  |  |  |  |  |
| 89 |  |  |  |  |  |  |

Table e11: Procalcitonin [ng/ml]; Data are median, its lower and its upper limit of 95% confidence interval (CI) of daily mean values of non-survivors and survivors at each day of stay

**Table e12: Patients with ECMO therapy on the respective day of stay**

| Day of stay | Non-Survivor | | Survivor | | p |
| --- | --- | --- | --- | --- | --- |
| 1 | 44 | 33.6% | 28 | 24.6% | 0.160 |
| 2 | 48 | 35.6% | 29 | 24.8% | 0.075 |
| 3 | 48 | 36.9% | 31 | 27.4% | 0.132 |
| 4 | 49 | 38.9% | 30 | 27.3% | 0.072 |
| 5 | 50 | 40.0% | 30 | 28.8% | 0.095 |
| 6 | 46 | 38.0% | 28 | 28.6% | 0.153 |
| 7 | 47 | 40.2% | 26 | 28.0% | 0.080 |
| 8 | 43 | 38.7% | 26 | 29.9% | 0.230 |
| 9 | 41 | 39.8% | 25 | 32.1% | 0.350 |
| 10 | 39 | 39.4% | 23 | 32.9% | 0.421 |
| 11 | 36 | 40.9% | 21 | 31.8% | 0.312 |
| 12 | 33 | 40.7% | 19 | 31.7% | 0.294 |
| 13 | 32 | 41.0% | 15 | 27.3% | 0.140 |
| 14 | 27 | 39.7% | 15 | 28.3% | 0.249 |
| 15 | 24 | 40.0% | 14 | 28.0% | 0.229 |
| 16 | 20 | 40.8% | 10 | 21.7% | 0.051 |
| 17 | 16 | 35.6% | 10 | 22.2% | 0.245 |
| 18 | 14 | 33.3% | 7 | 17.5% | 0.131 |
| 19 | 13 | 35.1% | 6 | 17.6% | 0.114 |
| 20 | 13 | 39.4% | 6 | 19.4% | 0.104 |
| 21 | 12 | 44.4% | 5 | 16.7% | 0.041 |
| 22 | 9 | 39.1% | 4 | 12.9% | 0.051 |
| 23 | 8 | 42.1% | 4 | 14.8% | 0.049 |
| 24 | 5 | 33.3% | 4 | 15.4% | 0.248 |
| 25 | 5 | 33.3% | 4 | 16.0% | 0.255 |
| 26 | 5 | 41.7% | 4 | 17.4% | 0.220 |
| 27 | 5 | 50.0% | 4 | 16.7% | 0.085 |
| 28 | 4 | 44.4% | 4 | 18.2% | 0.185 |
| 29 | 3 | 37.5% | 4 | 18.2% | 0.345 |
| 30 | 3 | 37.5% | 3 | 15.0% | 0.311 |
| 31 | 3 | 37.5% | 3 | 14.3% | 0.305 |
| 32 | 3 | 42.9% | 3 | 15.0% | 0.290 |
| 33 | 3 | 42.9% | 3 | 17.6% | 0.307 |
| 34 | 2 | 33.3% | 3 | 18.8% | 0.585 |
| 35 | 1 | 20.0% | 3 | 20.0% | 1 |
| 36 | 1 | 20.0% | 2 | 13.3% | 1 |
| 37 | 1 | 20.0% | 2 | 13.3% | 1 |
| 38 | 1 | 20.0% | 2 | 14.3% | 1 |
| 39 | 0 |  | 2 | 16.7% | 1 |
| 40 | 0 |  | 2 | 18.2% | 1 |
| 41 | 0 |  | 1 | 11.1% | 1 |
| 42 | 0 |  | 1 | 11.1% | 1 |
| 43 | 0 |  | 1 | 14.3% | 1 |
| 44 | 0 |  | 1 | 14.3% | 1 |
| 45 | 0 |  | 1 | 14.3% | 1 |
| 46 | 0 |  | 1 | 16.7% | 1 |

Table e12: Data are presented as total number of ECMO patients and relative proportion on each day of stay, and significance level (p).
